# Diamondoid ether clusters in helium nanodroplets†

**DOI:** 10.1039/d3cp00489a

**Published:** 2023-03-15

**Authors:** Jasna Alić, Roman Messner, Marija Alešković, Florian Küstner, Mirta Rubčić, Florian Lackner, Wolfgang E. Ernst, Marina Šekutor

**Affiliations:** a Department of Organic Chemistry and Biochemistry, Ruđer Bošković Institute Bijenička cesta 54 10 000 Zagreb Croatia msekutor@irb.hr; b Institute of Experimental Physics, Graz University of Technology Petersgasse 16 8010 Graz Austria wolfgang.ernst@tugraz.at florian.lackner@tugraz.at; c Department of Chemistry, Faculty of Science, University of Zagreb Horvatovac 102a 10 000 Zagreb Croatia

## Abstract

Diamondoid ethers were introduced into superfluid helium nanodroplets and the resulting clusters were analyzed by time-of-flight mass spectrometry. Clusters of higher abundances (magic number clusters) were identified and the corresponding potential cluster geometries were obtained from GFN2-xTB and DFT computations. We found that the studied diamondoid ethers readily self-assemble in helium nanodroplets and that London dispersion attraction between hydrocarbon subunits acts as a driving force for cluster formation. On the other hand, hydrogen bonding between ether oxygens and trace water molecules fosters the eventual breakdown of the initial supramolecular aggregate.

## Abstract 2

Self-assembly behavior of diamondoid ethers in helium nanodroplets is governed by interplay of London dispersion and hydrogen bonding.

## Graphical abstract

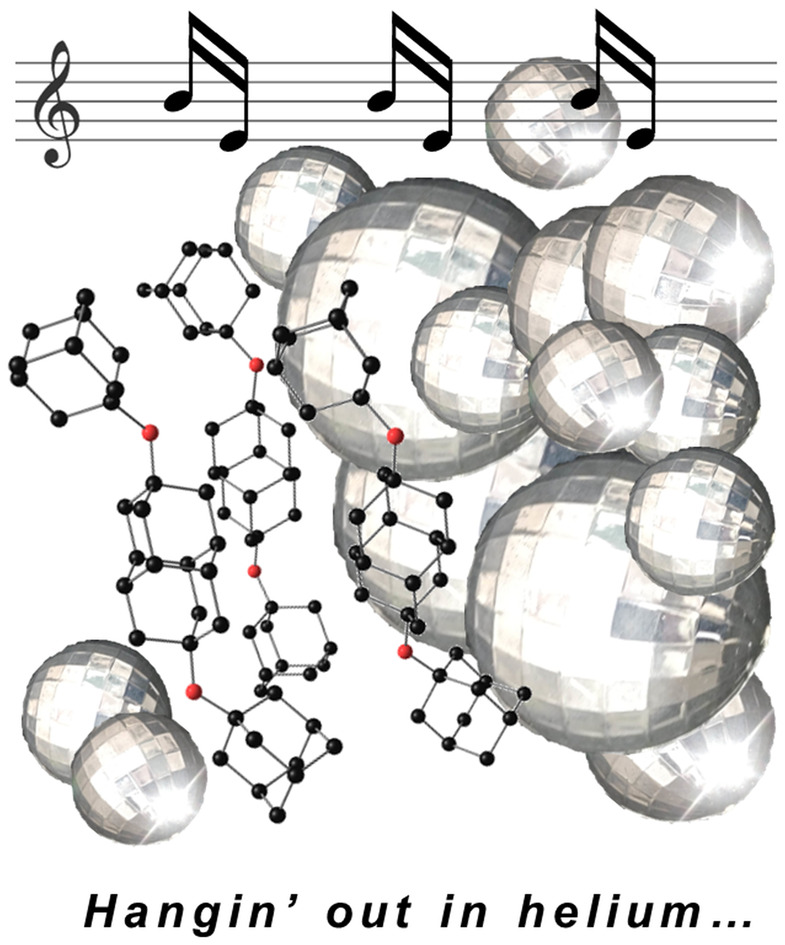

## Introduction

Nanostructure frameworks consisting of functionalized carbon-containing molecules may provide the basis for new materials with applications in areas ranging from materials science through bioengineering to medicine.^1,2^ However, preparation of molecular nanoparticles often includes the use of solution-based methods and the presence of solvent may have a significant impact on the non-covalent bonding interactions. On the other hand, assembly of molecules in superfluid helium nanodroplets (HNDs) has recently emerged as a suitable non-disruptive technique for the study of weak interactions between molecules. The expansion of gaseous helium into a high vacuum at cryogenic temperatures^3,4^ produces helium aggregates that can trap atoms and molecules which are picked up upon collision. Subsequently, various clusters can form during this entrapment process and HNDs are therefore essentially small reaction chambers.^5–13^ The binding energy released upon cluster formation is dissipated through the evaporation of helium atoms. As helium becomes superfluid at low pressures and near-zero Kelvin temperatures, properties like vanishing viscosity and high heat conductivity emerge,^14^ making the helium aggregates a suitable medium for trapping weakly binding complexes.^15,16^ For example, exceptional properties of HNDs were used to investigate molecular clusters like (V_2_O_5_)_*n*_,^9^ weakly bound alkali triplet-dimers^17^ and quartet-trimers,^18^ diamantane clusters,^19^*etc.* HNDs are therefore a promising matrix for the generation and analysis of self-organized clusters consisting of diamondoid molecules like adamantane^20,21^ and diamantane.^19^

Diamondoids belong to a class of naturally occurring,^22^ nanometer sized, cage-like hydrocarbons that are mostly superimposable on the cubic diamond lattice.^23,24^ As they are hydrogen-terminated, they can be selectively functionalized depending on the envisioned application.^24^ Another characteristic of diamondoids is their richness in C–H bonds, enabling their aggregation through London dispersion (LD)^25^ intermolecular interactions. Note that despite LD being inherently weak, it is additive in nature and many close contacts between molecules are needed for the emergence of an observable macroscopic effect which may be enhanced through many-body interactions. We previously explored the influence of LD interactions on diamondoid self-assembly but on solid surfaces, so our study was limited to only two dimensions as diamondoid molecules were deposited on planar carriers.^26–29^ HNDs on the other hand provide the means to expand our diamondoid investigation to a 3D environment.^19^ It should be mentioned that HNDs are also an excellent mimic of interstellar conditions due to achieved low temperatures and pressures, thus making exploration of diamondoid aggregates in HNDs important from the perspective of astrochemistry as their presence in space was also confirmed.^30,31^

While the properties of atomic clusters vary largely with their size and shape, clusters of large molecules offer many more parameters that contribute to their optical, electronic, or mechanical properties, such as molecular orbitals involved in the bonding, structural symmetries, packing effects, *etc.*^32^ In the case of diamondoid derivatives that possess functional groups capable of forming hydrogen bonds, it is expected that complex formation and stability is governed by both hydrogen bonding and dispersion interactions. Our current work has the goal to investigate the interplay of these interactions in complexes of diamondoid ether molecules (hydrogen bond acceptors in the presence of trace water molecules) which are formed in a clean molecule-by-molecule aggregation at low temperature inside a superfluid helium environment. In addition to gaining new fundamental insights into intermolecular relationships acting in these complexes, findings in this field have implications for future applicative use in designing new materials with desirable properties.

**Fig. 1:**
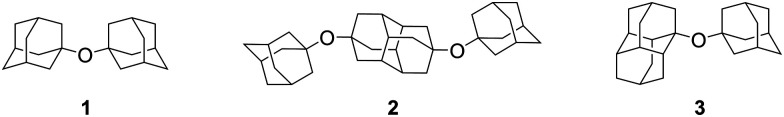
Structures of diamondoid ethers 1–3 used for HND cluster preparation.

As part of our investigation of covalent diamondoid assemblies consisting of two or more diamondoid cages connected with a heteroatom linker, we recently prepared a series of diamondoid ether derivatives.^29,33^ We explored on-surface assemblies and capability of monolayer formation in a 2D environment and demonstrated the importance of LD interactions acting in such supramolecular systems. Some ether derivatives^34^ were previously studied at HND conditions but as they were not as bulky as diamondoid ethers, they could not engage in numerous intermolecular C–H bond contacts that would produce a strong additive LD effect. In the scope of this study we therefore explored cluster formation of diamondoid ethers 1–3 (Fig. 1) in HND conditions. The experimental findings were supported by a computational analysis, with emphasis on the identified cluster sizes with large abundances, *i.e.*, the magic numbers. Our computational approach was described previously^19^ and applied here for the structural analysis of the clusters. To the best of our knowledge, aggregation behavior of diamondoids in HNDs has only been studied for adamantane and diamantane, other diamondoids or their derivatives have remained unexplored up to now.

## Experimental

### Synthesis

1,1′-Diadamantyl ether (1) and 1-adamantyl-1-diamantyl ether (3) were synthesized according to previously published procedures,^29,33,35^ while the preparation of 4,9-bis(1-adamantyloxy)diamantane (2) is first described here (*vide infra*).

Nuclear magnetic resonance (^1^H and ^13^C NMR) spectra were recorded with Bruker AV-300 or AV-600 NMR spectrometers and the NMR spectra were referenced to the residual proton or carbon signal of the used deuterated solvent as an internal standard. Infrared (IR) spectra were recorded with a FT-IR ABB Bomem MB 102 or FT IR-ATR PerkinElmer UATR Two spectrometers (range 400 to 4000 cm^−1^). Matrix assisted laser desorption/ionization – time of flight mass spectra (MALDI-TOF MS) were obtained in “reflectron” mode with an Applied Biosystems Voyager DE STR instrument (Foster City, CA). Determination of melting points was performed by differential scanning calorimetry (DSC) analysis using Simultaneous Thermal Analyzer (STA) 6000 (PerkinElmer, Inc.) in a platinum crucible with cap (non-hermetically sealed). Around 5 mg of each sample were analyzed using a 10 °C min^−1^ heating rate and under a 20 mL min^−1^ nitrogen gas flow. Obtained data was processed with the Pyris Data Analysis software. Gas chromatography–mass spectrometry (GC–MS) analyses for the reaction monitoring were performed on an Agilent 7890B/5977B GC/MSD instrument equipped with a HP-5ms column. Thermogravimetric analyses were performed with a Mettler-Toledo TGA/DSC 3+ instrument using 70 μL Al_2_O_3_ crucibles with lids. All experiments were performed in a dynamic nitrogen atmosphere with a flow rate of 50 cm^3^ min^−1^ and the heating rate of 5 K min^−1^. All of the solvents were obtained from commercial sources and used without further purification. Diamantan-4,9-diol^36^ and 1-adamantyl methanesulfonate^35^ were synthesized according to literature procedures.
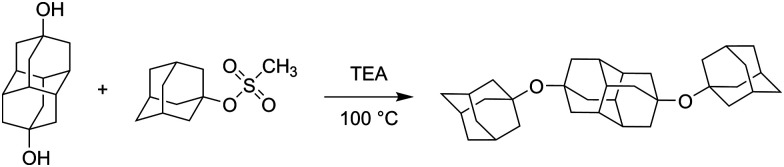


Preparation of 4,9-bis(1-adamantyloxy)diamantane (2): in a glass vessel (2–5 ml) with a Teflon stopper diamantan-4,9-diol (110 mg, 0.5 mmol), 1-adamantyl methanesulfonate (576 mg, 2.5 mmol, 5 equiv.) and triethylamine (0.38 ml, 2.7 mmol, 5.5 equiv.) were added and the reaction mixture was heated to 100 °C with magnetic stirring and kept at that temperature for 3 days. After the reaction mixture was cooled to room temperature, CH_2_Cl_2_ (30 ml) and water (20 ml) were added and the layers were separated. The solid that remained in the water phase was filtered using a sintered funnel (porosity 5), washed with water, *n*-hexane, CHCl_3_ and methanol and dried on air, yielding 2 as a white powder (150 mg, 61%).

Analytical characterization of ether 2: m.p. 399–401 °C. ^1^H NMR (600 MHz, toluene-d_8_, 70 °C): *δ* = 2.03 (bs, 6H), 1.96–1.93 (m, 12H), 1.92 (s, 12H), 1.75 (s, 6H), 1.61–1.54 (m, 12H). ^13^C NMR (75 MHz, toluene-d_8_, 70 °C): *δ* = 74.1 (C, C–O, 2C), 73.1 (C, C–O, 2C), 46.4 (CH_2_, 6C), 45.8 (CH_2_, 6C), 39.7 (CH, 6C), 37.0 (CH_2_, 6C), 31.6 (CH, 6C). IR (neat), *ν*_max_/cm^−1^: 2904 (s), 2889 (s), 1444 (w), 1341 (m), 1247 (w), 1119 (m), 1092 (s), 1083 (s), 1029 (m), 898 (w), 615 (w). HRMS (MALDI): calcd. for [C_34_H_48_O_2_]^+^ 488.3654; found 488.3650.

### Helium nanodroplets

The apparatus used for the generation of the HNDs is described in detail in ref. 9 and 10. In short, pressurized high purity He (99.9999%) is cooled to temperatures below 20 K by a closed-cycle refrigerator (Sumitomo RDK-408D2) and expanded through a 5 μm nozzle into high vacuum. During this process the gaseous He condenses into small superfluid droplets. At the expansion conditions used in the experiments (*p*_He_ = 60 bars, *T*_He_ = 11.5–12.5 K) He droplets with a mean diameter of 40 to 60 nm, consisting of about 1 × 10^6^ to 3 × 10^6^ He atoms, are formed.^3,4^ Subsequently, the beam passes a skimmer and the helium droplets pick up the desired dopant species in a separately pumped chamber. Here, we dope the droplets with synthesized diamondoid ethers 1–3 (compounds 1 and 3 were previously described^29,33,35^ and compound 2 was prepared using a similar procedure, *vide supra*) using a heated gas-pickup cell (100 °C), which is connected to a heated reservoir (70 °C) *via* a precision leak valve. The doped He droplets then enter the differentially pumped analysis chamber, where a reflectron time-of-flight mass spectrometer KAESDORF RTF50 is utilized to record mass spectra. Upon electron impact ionization ether cluster ions are expelled from the He droplets and can be detected at the corresponding mass channel. The employed emission current (*I*_em_) and ionization energy (*E*_el_) are *I*_em_ = 6.8 μA and *E*_el_ = 90 eV, respectively.

### Theoretical methods

Geometry optimizations of 1,1′-diadamantyl ether (1), 4,9-bis(1-adamantyloxy)diamantane (2) and 1-adamantyl-1-diamantyl ether (3) were performed with the Orca 5.0.3 program package^37,38^ using the B3LYP-gCP-D3(BJ)-ABC/def2-TZVPP level of theory,^39,40^ and the obtained minima were verified by frequency computations. Search for favourable ether cluster structures was done using the Conformer-Rotamer Ensemble Sampling Tool (CREST) based on the GFN (Geometries, Frequencies and Non-covalent interactions) methods^41,42^ by applying the iterative meta-dynamic (iMTD) sampling for non-covalently bound (NCI) complexes, clusters or aggregates (NCI-iMTD mode). The starting geometries for the CREST screening were taken from the corresponding X-ray crystal structures when available (Cambridge Crystallographic Data Center (CCDC) deposition numbers: 2156453 (1) and 2156490 (3)).^29^ Single point computations on the identified best cluster structures were performed using the B3LYP-gCP-D3(BJ)-ABC/def2-TZVPP level of theory. The choice of the density functional theory (DFT) method was based on our previous benchmarking of similarly bulky molecules in HNDs.^19^ We found that the use of Grimme's dispersion correction with Becke-Johnson damping (D3(BJ)),^39,40^ the three-body dispersion contributions term implemented in Orca as well as geometrical counterpoise (gCP) correction^43^ is desirable in order to account for subtle intermolecular interactions and mitigate the basis-set superposition errors (BSSE), respectively. Zero-point vibrational energy used during the assessment of cluster binding energy was obtained from the semi-empirical quantum mechanical GFN2-xTB method^44,45^ at 0.4 K to account for the temperature of the liquid He environment. Non-covalent interactions (NCI) plots were constructed using Multiwfn 3.6^46^ and visualized by Visual Molecular Dynamics (VMD) software.^47^

## Results and discussion

In previous studies of self-organized HND aggregates consisting of diamondoid molecules like adamantane^20,21^ and diamantane^19^ certain preferences for cluster abundances were found that belonged to particularly stable geometries. These observed privileged abundances, termed magic numbers, amounted to 13, 19, 38, 52, *etc.*, with the numbers corresponding to the number of individual molecules in the cluster. It was previously proposed that the first magic number 13 occurs because entropy and sphere-like shapes usually suffice for icosahedron formation.^48^ However, adamantane and diamantane molecules possess different molecular symmetries (*T*_d_ and *D*_3d_, respectively), suggesting that the cause for the formation of cluster with similar abundancies is not so clear cut. Additionally, intermolecular dispersion attraction between bulky hydrocarbons has a pronounced effect on supramolecular binding and cluster formation in the HNDs. We were therefore interested in exploring how a change in molecular symmetry affects the aggregation of molecules in a HND medium, unimpeded by the effect of common solvents. Additionally, we also wished to explore what happens when the organic molecule is no longer a pure hydrocarbon. With that in mind, we prepared diamondoid ether derivatives 1–3 (Fig. 1). Ether 1 consists of two adamantane subunits, bis-ether 2 possess a central diamantane subunit apically substituted with two adamantane scaffolds, and ether 3 consists of a diamantane subunit medially substitute with one adamantane. Structurally speaking, ethers 1 and 2 can essentially be described as looking like a shorter and a longer rod, respectively, and ether 3 is reminiscent of a T-shape.

**Fig. 2:**
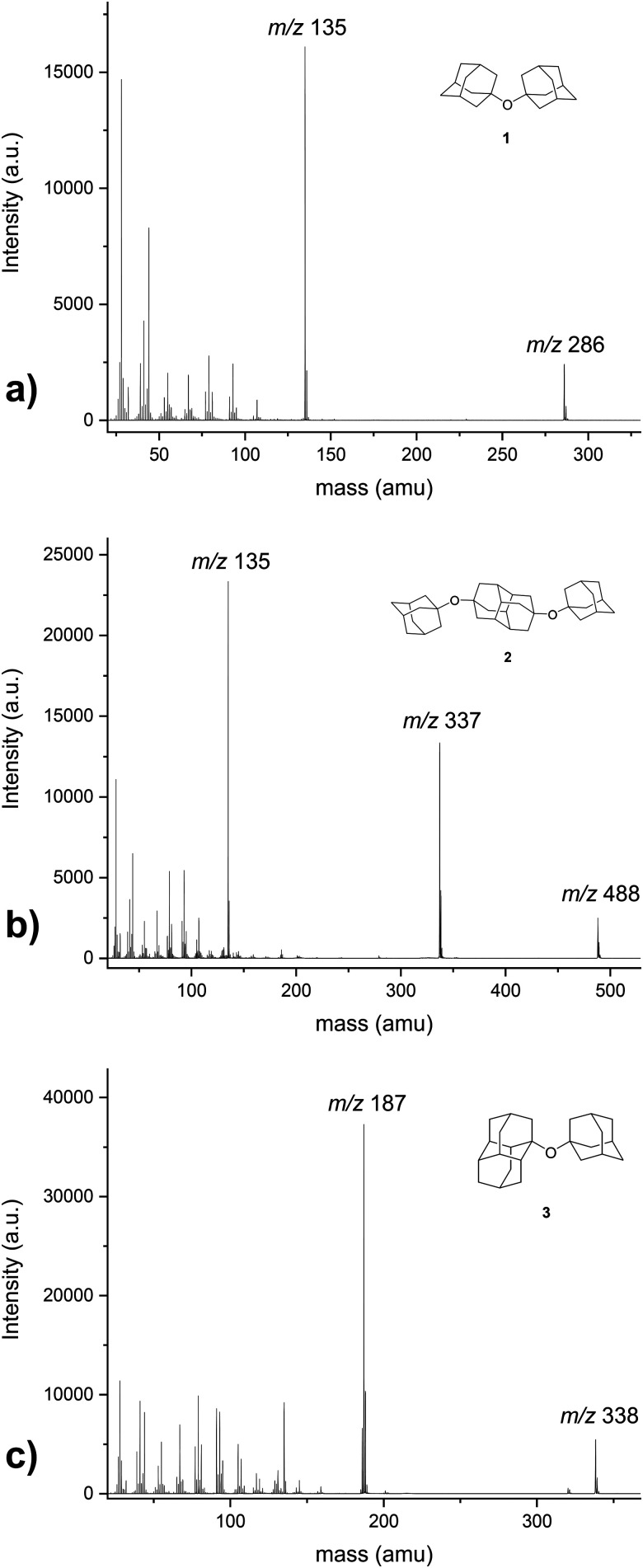
Recorded mass spectra of (a) 1,1′-diadamantyl ether (1), (b) 4,9-bis(1-adamantyloxy)diamantane (2), (c) 1-adamantyl-1-diamantyl ether (3). The spectra were acquired with the helium droplet beam blocked, *i.e.*, they correspond to mass spectra of bare gas phase molecules.

Fig. 2 presents mass spectra for ether samples, evaporated in the pickup chamber of the helium droplet machine with blocked helium droplet beam. In this case, ether clusters are not formed and in the absence of the helium matrix the mass spectra are governed by fragmented molecules. In all obtained mass spectra the respective molecular ion peak is present (*m*/*z* 286 for ether 1, *m*/*z* 488 for ether 2 and *m*/*z* 338 for ether 3) as well as the fragmentation peak characteristic for the appended diamondoid cage subunit that is present in the respective ether molecule (*m*/*z* 135 for 1 and 2 (adamantyl) and *m*/*z* 187 for 3 (diamantyl)). For bis-ether 2 there is also an additional prominent peak that corresponds to the loss of one adamantyl-O fragment (*m*/*z* 337).

**Fig. 3:**
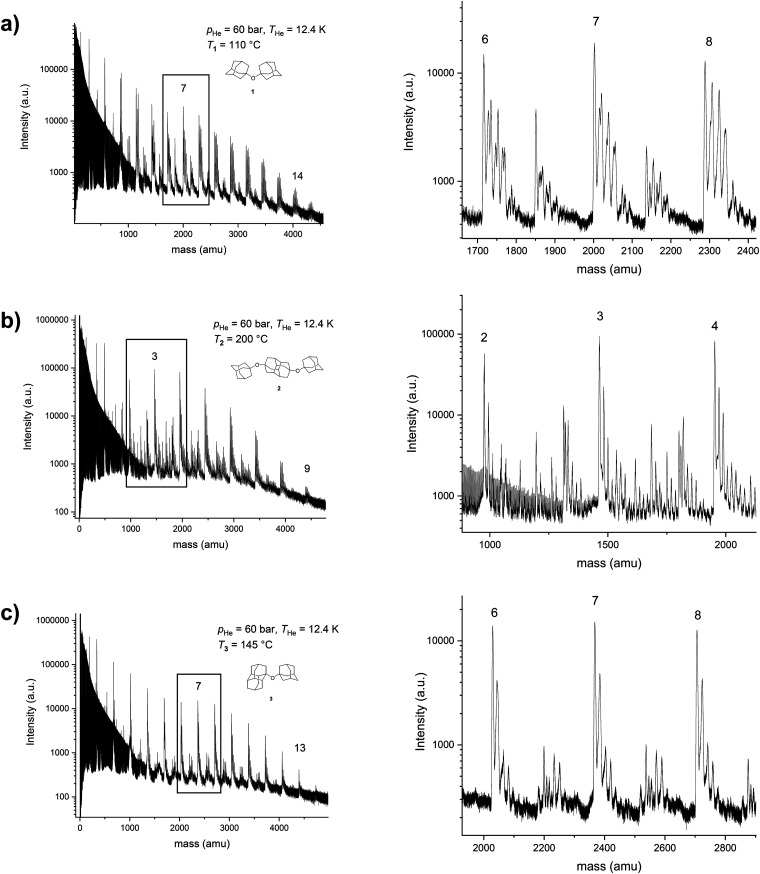
Recorded mass spectra of He droplets doped with (a) 1,1′-diadamantyl ether (1), (b) 4,9-bis(1-adamantyloxy)diamantane (2), (c) 1-adamantyl-1-diamantyl ether (3). *T*_He_ = 12.4 K, *p*_He_ = 60 bar. Enlarged areas around magic number peaks are depicted in panels to the right of the overview spectra for each ether species.

Mass spectra for the studied ethers 1–3 after being embedded in the HNDs are presented in Fig. 3. Questions about the different fragmentation behavior of molecules in the gas phase or inside helium droplets upon electron impact ionization were addressed in reference^9^ for vanadium oxide clusters; the corresponding experiments were carried out in the same laboratory as the current work. While gas phase vanadium pentoxide showed strong fragmentation already at electron impact ionization with 20 eV electrons and even more so for 89 eV, only very little fragmentation was observed when the molecules were surrounded by helium.^9^ Results for perfluoroethers in helium droplets obtained by the Scheier group^34^ nicely demonstrate fragmentation differences when the clusters are surrounded by helium since the HND medium has a strong effect on ion formation following electron impact ionization by proceeding *via* a charge hopping model inside the droplets. Gas phase mass spectra of the studied perfluoroethers contained only signals of fragments and no ions of the original gas phase molecules were observed.^34^ In contrast, the reported droplet mass spectra revealed formation of the molecular ion as well as a tendency towards formation of higher-mass fragment ions, when compared to the corresponding gas phase spectra of the respective perfluoroether. In our case the situation is slightly different since diamondoid ethers 1–3 readily produce the corresponding molecular ion peaks in their compound gas phase spectra (Fig. 2). In their droplet mass spectra many peaks besides the original molecules appear that correspond to a number of stabilized fragmented species or fragment combinations (Fig. 3). For example, in addition to the peaks indicative of ether clusters, peaks that combined different molecular fragments, parent ether molecules and/or water molecules are also present in a plethora of conglomerates with varying stability.

**Fig. 4:**
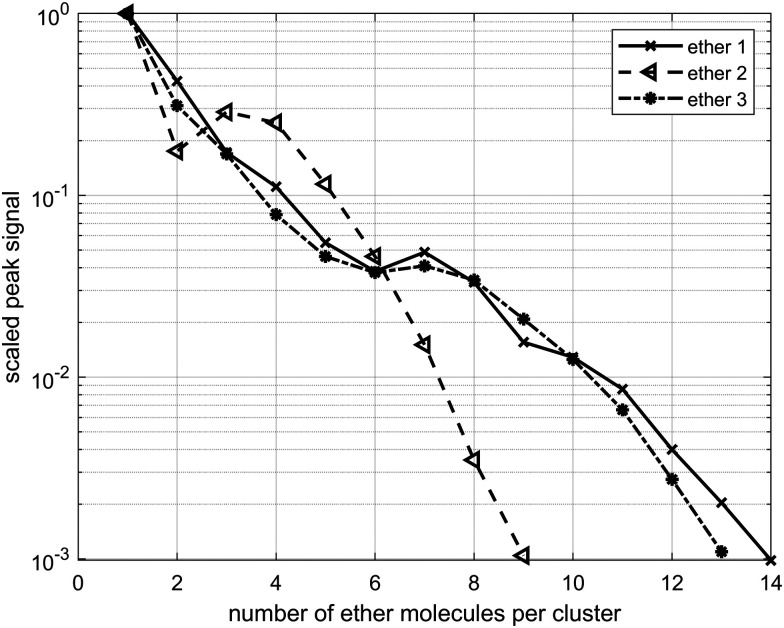
Cluster abundance for ethers 1–3. Clusters with *n* = 7 units of 1, *n* = 3 units of 2 and *n* = 7 units of 3 show a higher abundance, suggesting increased stability. Designation n refers to the number of ether molecules per cluster.

Each ether cluster peak is accompanied by several additional peaks as a consequence of residual water pickup at the background pressure of 10^−7^ mbar in the pick-up chamber.^9^ In Fig. 4 cluster abundance for ethers 1–3 is depicted, exhibiting magic numbers *n* = 7 for ethers 1 and 3 and *n* = 3 for ether 2.

**Fig. 5:**
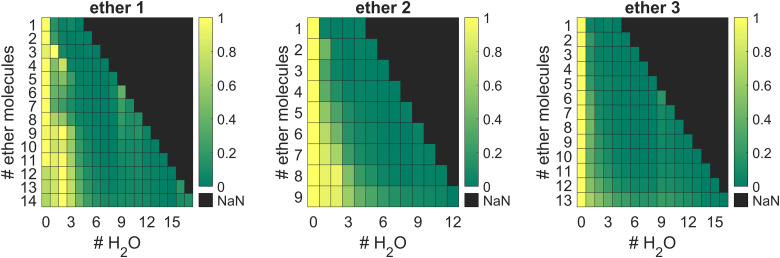
Amount of water molecules for clusters formed from ethers 1–3.

Ether clusters can uptake water molecules inside their aggregates (Fig. 5), ultimately leading to parent cluster erosion. Since water molecules eventually form their own hydrogen bonded clusters, they effectively destroy weaker LD networks acting between ether moieties. The origin of these trace water molecules is probably from pick-up of rest gas H_2_O during the flight of doped droplets through the apparatus and not from the initial ether samples. Namely, the results of thermogravimetric analyses (TGA) of the studied ethers (details in the ESI,† Fig. S2) confirmed that the samples did not contain any left-over water (crystal water), which could originate from synthetic protocols used to obtain the compounds in high purity. This finding comes as no surprise since ethers 1–3 are overall quite lipophilic and fail to incorporate water molecules inside their crystals when obtained from low polarity solvents (CCDC deposition numbers: 2156453 (1, obtained from *n*-pentane) and 2156490 (3, obtained from *n*-hexane)).^29^ The results of thermal analyses also corroborate that no decomposition occurs at temperatures used for heated pickup procedure, *i.e.,* at temperatures when the ether samples were introduced into the HND generating apparatus.

**Fig. 6:**
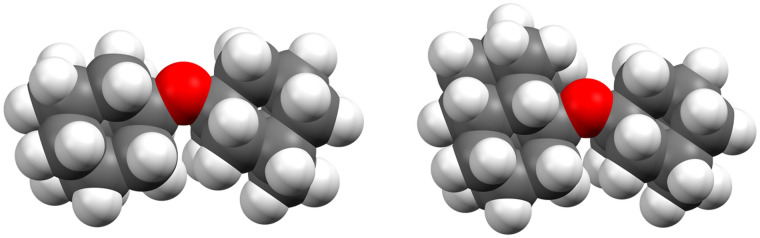
Space-filling models of ethers 1 (left) and 3 (right), structures obtained from computations (*vide infra*).

Curiously, the observed clusters of ether 3 contain noticeably less water than the other two ether clusters (Fig. 5). This difference in water molecule abundance can be rationalized by the structural features of the ethers in question. More specifically, in the structure of ether 3 the oxygen atom, which is expected to partake in the hydrogen bond formation with the surrounding water molecules, is spatially much more shielded with the appended hydrocarbon cages than it is the case for ether 1 (Fig. 6). As a consequence, the initial approach of a water molecule to the ether oxygen is disfavored and ultimately leads to conglomerates with negligible water content. On the other hand, oxygen incorporated into ether 1 is much more available for intermolecular interaction with water and the resulting cluster is therefore richer in water content. However, as mentioned previously, this has a detrimental effect on ether cluster stability since water molecules start destroying the initial supramolecular organization. The same trend was observed for ether 2 that is also structurally available to water molecules like ether 1 but can incorporate even more water content seeing as it has two oxygens that serve as hydrogen bond formation sites.

To gain more structural insight into the arrangement of diamondoid ether molecules 1–3 in HNDs, we performed a computational analysis of the three experimentally most abundant clusters, CL1 (*n* = 7), CL2 (*n* = 3) and CL3 (*n* = 7). Due to the size of the system and many existing degrees of freedom, we performed a search for viable cluster structures using CREST based on the GFN methods,^41,42^ more specifically, by applying the iterative meta-dynamic sampling for non-covalently bound systems (NCI-iMTD mode). After identifying the best candidates, we did single point computations on those geometries using the B3LYP-gCP-D3(BJ)-ABC/def2-TZVPP level of theory. Our previous benchmarking for similar systems provided us with a reliable DFT method that gave reasonably accurate binding energy values and we identified D3(BJ) dispersion correction,^39,40^ three-body dispersion contributions term implemented in Orca as well as gCP correction^43^ to be important for accounting of subtle intermolecular interactions and for avoidance of the BSSE.

**Fig. 7:**
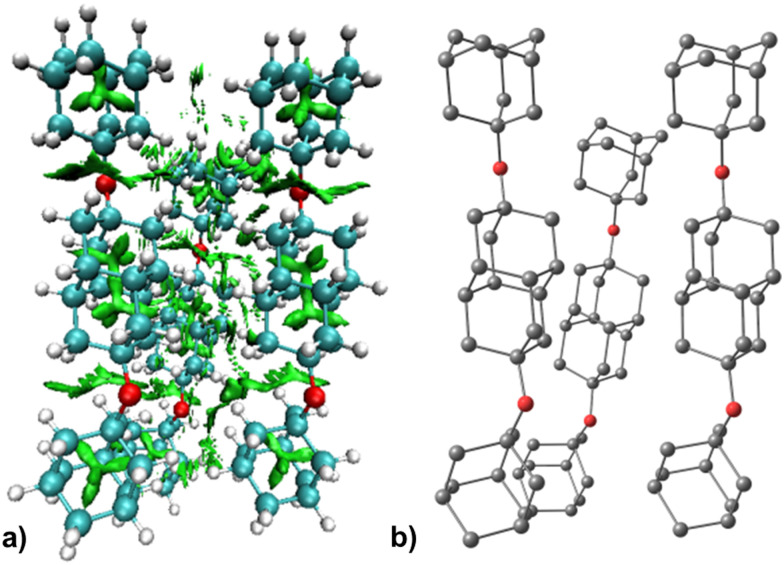
Non-covalent interactions (NCI) plot of the computed structure of CL2 with attraction areas depicted in green (left) and the corresponding molecules of ether 2 depicted without hydrogen atoms for clarity (right).

The obtained interaction energies (Table S2, ESI†) for ether clusters CL1–CL3 are in line with our previously obtained values for diamantane clusters^19^ and confirm that aggregation of diamondoid ethers in HNDs is indeed an energetically favorable process. As intermolecular LD appears to be the main driving force for the cluster aggregation, increase in cluster size leads to the rise of interaction energies due to multiplication of intermolecular close contacts. For example, areas of dense close contacts between molecules of ether 2 are depicted in green in Fig. 7. The computed stable cluster structure of CL2 consists of three ether moieties that are almost parallel in their orientation. Such spatial arrangement reflects the tendency to maximize the number of close contacts between the C–H bonds, a behavior typical for LD driven supramolecular structures. On the other hand, both ethers 1 and 3 form clusters consisting of seven molecules but adopt a less ordered shape (Fig. S3, ESI†). Close contacts between ether molecules are again of importance for aggregation but fail to produce a higher structural order, probably due to a less rod-like shape of molecules 1 or 3. A pronounced overall difference of cluster shapes becomes apparent when comparing a spherical diamondoid adamantane with diamondoid ethers. Namely, sphere-like molecules apparently pack much more symmetrically in the available space and magic number abundances are more prominent. On the other hand, more flexible ethers 1–3 cannot produce a more ordered environment during packing as they are essentially limited with their shape but also with additional degrees of freedom. Their rotation around the ether C–O bonds provides them with more flexibility to “hunt” for close contacts with their cage subunits at the expense of the overall cluster shape. At first glance ether 2 is an outlier that forms an aggregate with only three molecules but after considering its structural features it becomes apparent that its elongated backbone spanning throughout the whole molecule actually enables preferential intermolecular side interactions as a means of maximizing close contacts. Three ether units in a cluster are therefore a good balance between numerous LD contacts and a small number of interacting molecules that strive to aggregate together.

Experimentally observed magic numbers differ when comparing diamondoid hydrocarbons and diamondoid ethers. We previously proposed that the influence of starting packing in the helium environment can be responsible for observed magic number clusters of diamondoid hydrocarbons but this obviously breaks down when structurally more complex molecules are introduced to the HND system. In principle, a shell of helium surrounding the first molecular dopant inside the droplet may create a barrier against the bond formation with the next incoming molecule, but the arrangement of such a helium shell is held together only by weak He^…^He interactions that obviously cannot compete with the disruption caused by the introduction of further ether molecules. Only in the case of such weak interaction as for alkali-rare gas diatomics (*e.g.*, Rb–Xe in ref. 16), the helium environment prevents aggregation. Despite being shielded on both sides by a bulky hydrocarbon cage subunit, the ether oxygen still exerts a strong enough influence on its surroundings and breaks down the existing helium networks. Addition of subsequent ether molecules during cluster generation enables a favorable ether–ether interaction and only solidifies the structural breakdown of the helium environment around the dopants. Coupled with a flexibility of the C–O bond and a non-spherical shape of the scaffolds, these molecular features ultimately result in qualitatively different and quantitatively lower abundancies of the observed magic number clusters.

## Conclusions

We studied aggregation of diamondoid ethers 1–3 in superfluid helium nanodroplets and found significant differences in formed magic number clusters when compared to previously explored diamondoid hydrocarbons. Thus, magic number clusters CL1 (*n* = 7), CL2 (*n* = 3) and CL3 (*n* = 7) were successfully identified and characterized. Our experimental findings are supported by GFN2-xTB and DFT computations and gave rise to potential cluster geometries. London dispersion interaction between the cages is identified as a driving force for supramolecular assembly and the presence of ether oxygens most likely disrupts the initially ordered helium environment. Since ether oxygens are good hydrogen bond acceptors, traces of water molecules enable hydrogen bond formation and ultimately lead to the degradation of the starting ether cluster. Such subtle effects demonstrate the precarious balance existing during the interplay of different interaction modes, especially since the other hydrogen bond partner (water) is present only sparingly. However, when oxygen atoms are sterically shielded with diamondoid subunits, making hydrogen bond formation challenging, the resulting cluster has overall lower water content. This finding suggests that structural design of cluster-forming molecules could provide a way to control stability of clusters that are held together with weak intermolecular forces.

## Author contributions

The manuscript was written through contributions of all authors. All authors have given approval to the final version of the manuscript.

## Conflicts of interest

There are no conflicts to declare.

## Supplementary Material

CP-025-D3CP00489A-s001
