# Supplementary material for: Diamondoid ether clusters in helium nanodroplets
Source: Phys Chem Chem Phys. 2023 Mar 15;25(17):11951–8. doi: 10.1039/d3cp00489a (PMC10155488; doi:10.1039/d3cp00489a)
Supplement: CP-025-D3CP00489A-s001 [file CP-025-D3CP00489A-s001.pdf]

Electronic Supplementary Material (ESI) for Physical Chemistry Chemical Physics.
This journal is © the Owner Societies 2023

Supporting information

Diamondoid ether clusters in helium nanodroplets

Jasna Alić,[a, ‡] Roman Messner,[b, ‡] Marija Alešković,[a] Florian Küstner,[b] Mirta Rubčić,[c]

Florian Lackner[b,*] Wolfgang E. Ernst,[b,*] Marina Šekutor[a,*]

[a] Department of Organic Chemistry and Biochemistry, Ruđer Bošković Institute, Bijenička cesta 54, 10 000 Zagreb, Croatia, msekutor@irb.hr

[b] Institute of Experimental Physics, Graz University of Technology, Petersgasse 16, 8010 Graz, Austria, wolfgang.ernst@tugraz.at, florian.lackner@tugraz.at

[c] Department of Chemistry, Faculty of Science, University of Zagreb, Horvatovac 102a, 10 000 Zagreb, Croatia

[‡]Both contributors are considered first authors.

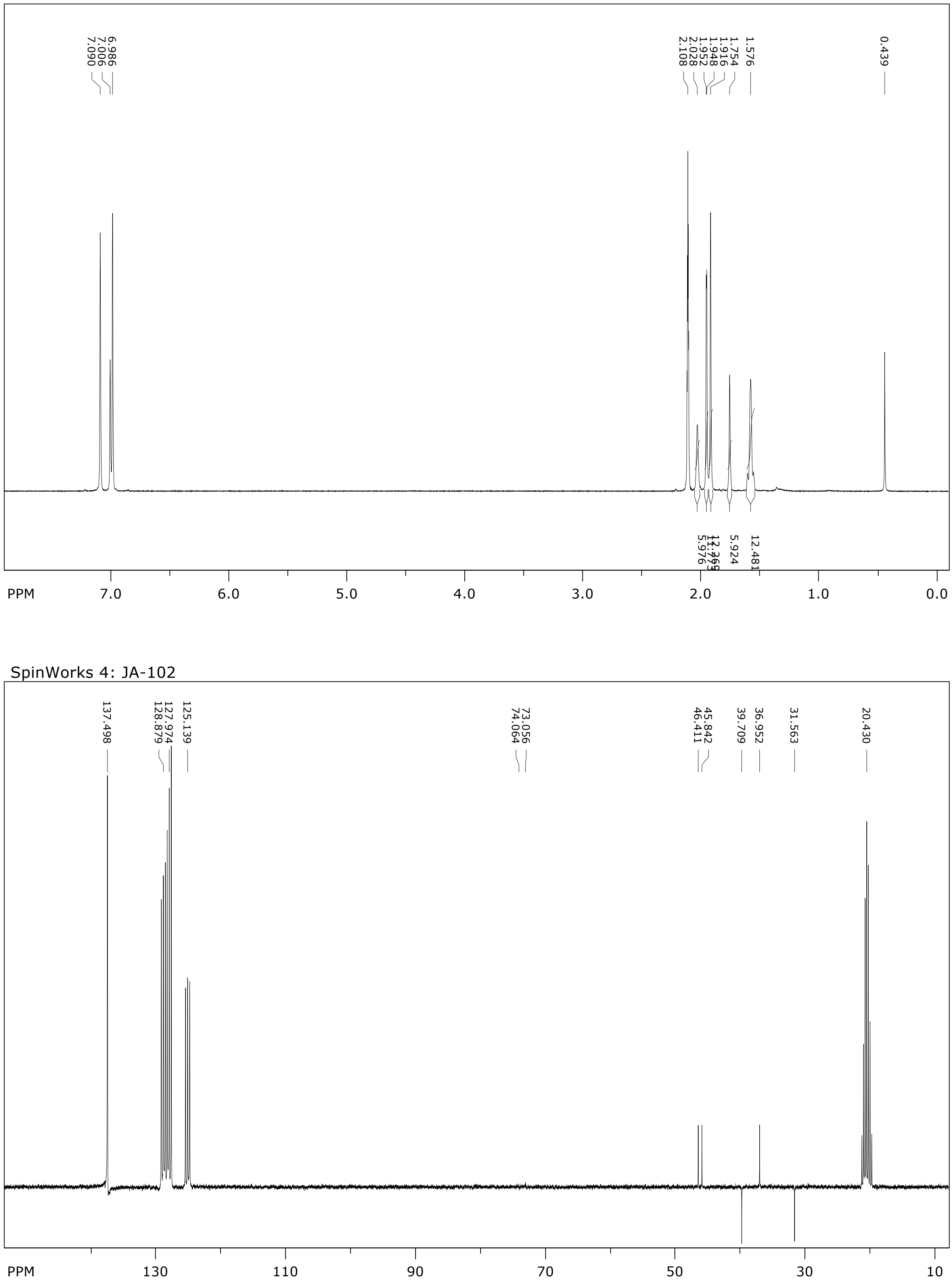

Figure S1. ^{1}H (600 MHz, toluen-d_8, 70 °C) and ^{13}C NMR (75 MHz, toluen-d_8, 70 °C) spectra of 4,9-bis(1-adamantyloxy)diamantane (**2**).

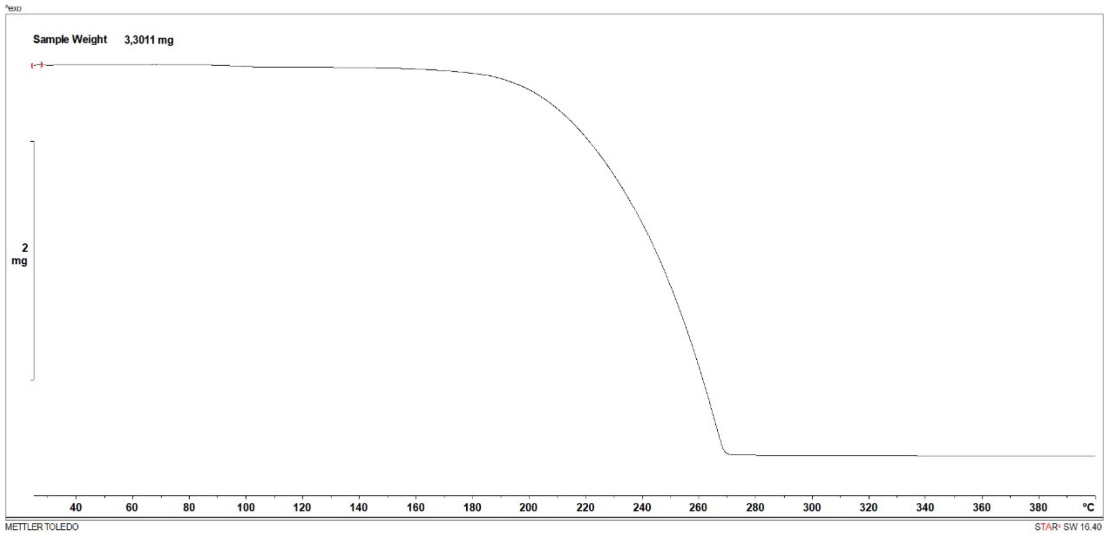

a)

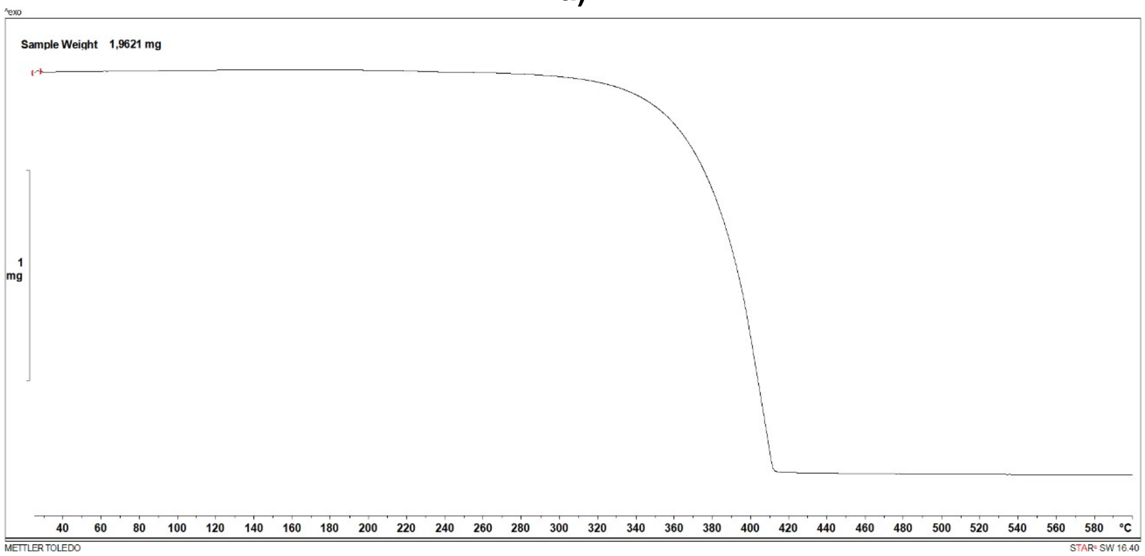

b)

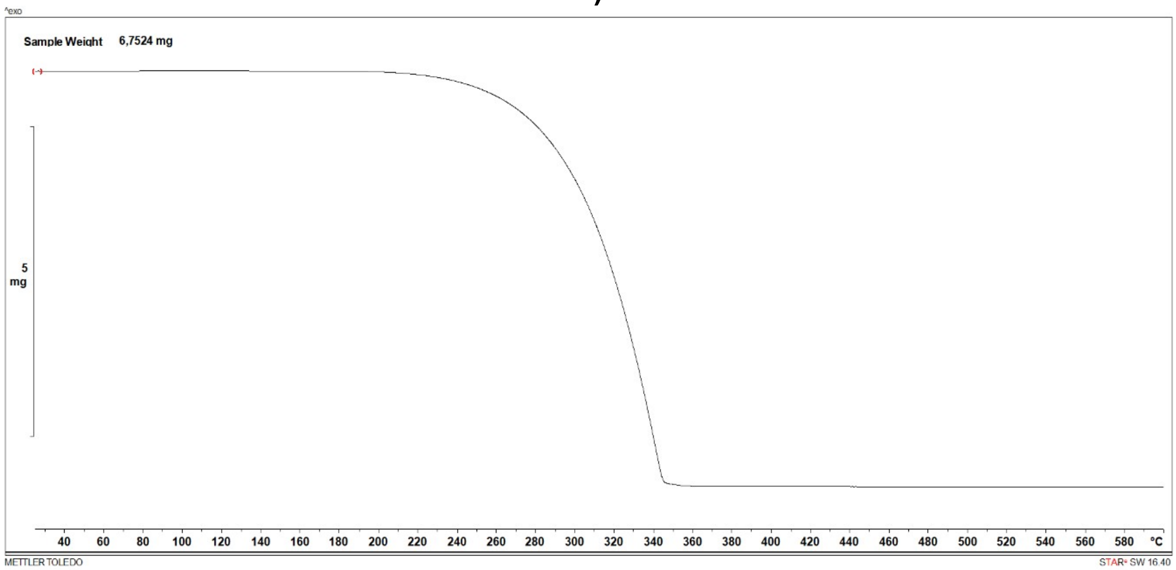

c)

Figure S2. TG curves for ethers: (a) **1**, (b) **2**, (c) **3**.

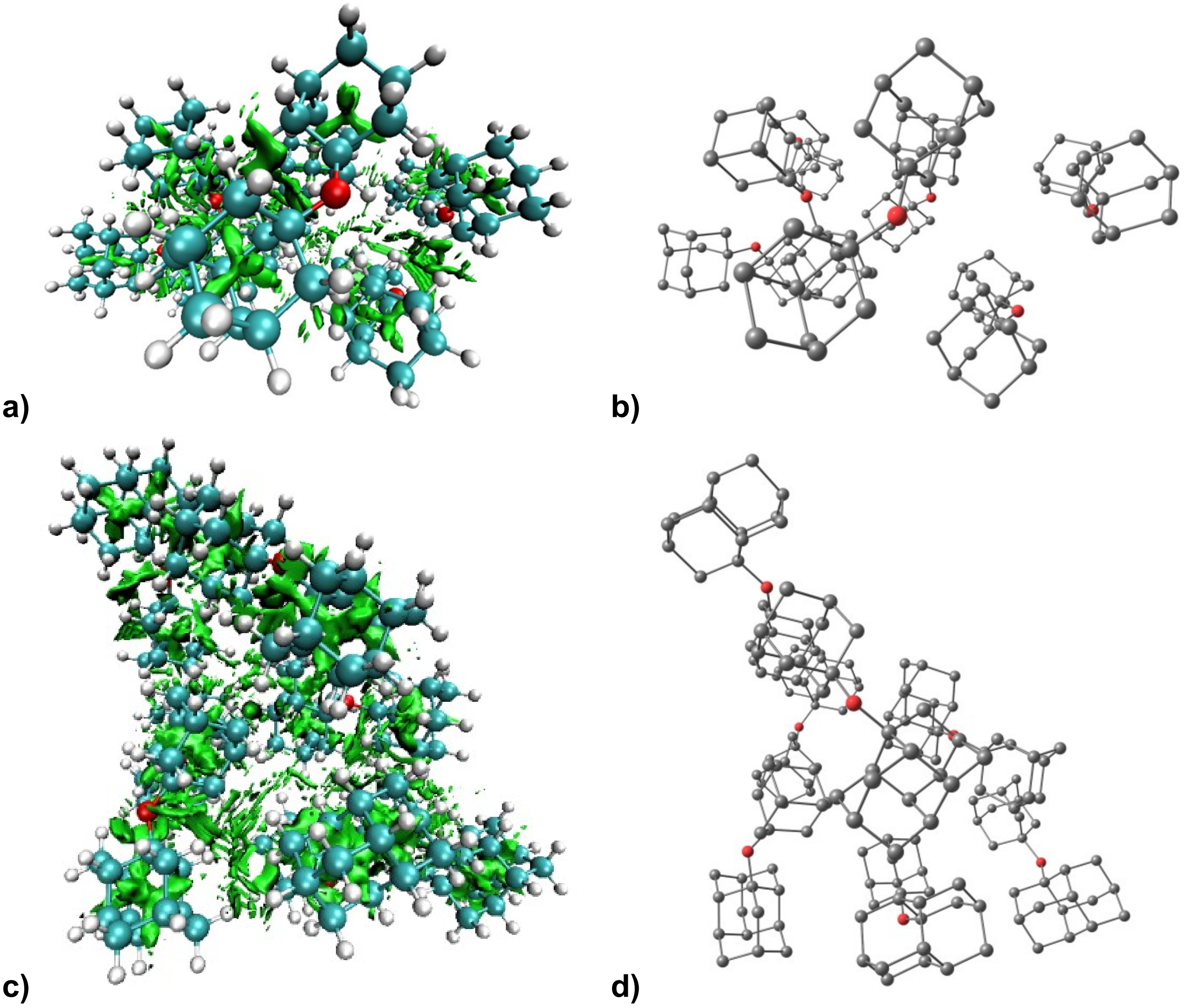

Figure S3. NCI plots of the computed structure of **CL1** (a) and **CL3** (c) with non-covalent interactions depicted in green, and the corresponding molecules building **CL1** (b) and **CL3** (d) depicted without hydrogen atoms for clarity.

Table S1. Electronic energies, zero-point vibrational energies, enthalpies and Gibbs energies of ethers **1**–**3** and clusters **CL1**–**CL3** in Hartree computed using the GFN2-xTB method at 0.4 K.

compound	*E*	*ZPVE*	*H*	*G*
**1**	-62.330173	0.462195	-61.867972	-61.867986
**2**	-105.676053	0.755918	-104.920130	-104.920144
**3**	-72.982695	0.533355	-72.449334	-72.449349
**CL1**	-436.399649	3.250816	-433.148827	-433.148800
**CL2**	-317.071649	2.277312	-314.794332	-314.794326
**CL3**	-510.975191	3.751655	-507.223531	-507.223503

Table S2. Electronic energies of ethers **1–3** and clusters **CL1–CL3** in Hartree and interaction energies, ΔH(0 K),[a] for clusters **CL1–CL3** in kcal mol^{-1} computed at the B3LYP-gCP-D3(BJ)-ABC/def2-TZVPP level of theory.

compound	*E*	compound	*E*	ΔH
**1**	-855.263900	**CL1**	-5986.934860	-45.2
**3**	-1474.722022	**CL2**	-4424.206671	-19.5
**3**	-1010.080285	**CL3**	-7070.655192	-47.1

[a] Interaction energies are defined as a difference between the energy of the cluster and the energy of the corresponding number of ether molecules. *ZPVE* taken from GFN2-xTB computations.

Table S3. Geometries of ethers **1–3** and clusters **CL1–CL3** in Cartesian coordinates in Å computed using the GFN2-xTB method at 0.4 K.

**1**			
6	-1.864116000	3.764963000	0.629218000
6	-2.697168000	2.563435000	1.101225000
6	-1.756718000	1.448260000	1.578255000
6	-0.940470000	3.323230000	-0.515147000
1	-1.270526000	4.159761000	1.459397000
1	-2.523024000	4.571493000	0.292337000
6	-0.004294000	2.203955000	-0.028074000
6	-0.824317000	0.987380000	0.444531000
1	-2.326243000	0.584744000	1.931443000
1	-1.140206000	1.790857000	2.413089000
1	0.614454000	2.548590000	0.804839000
1	0.668403000	1.920605000	-0.836858000
6	-3.550255000	2.043335000	-0.065681000
1	-3.347280000	2.865256000	1.927379000
6	-1.695091000	0.475833000	-0.715999000
6	-2.627183000	1.599043000	-1.209259000
1	-4.230822000	2.826670000	-0.413691000
1	-4.169755000	1.203619000	0.264415000
1	-2.286701000	-0.377595000	-0.376398000
1	-1.072480000	0.135912000	-1.545417000
6	-1.787251000	2.794949000	-1.684294000
1	-0.338685000	4.172512000	-0.851509000
1	-1.136642000	2.492162000	-2.511021000
1	-2.440911000	3.585872000	-2.064971000
1	-3.227825000	1.218786000	-2.040575000
8	-0.000001000	0.000000000	1.079435000
6	0.824315000	-0.987381000	0.444532000
6	1.756716000	-1.448260000	1.578256000
6	2.697168000	-2.563433000	1.101227000
1	1.140204000	-1.790858000	2.413090000
1	2.326240000	-0.584743000	1.931445000
6	1.695089000	-0.475834000	-0.715999000
6	2.627183000	-1.599042000	-1.209257000
1	2.286697000	0.377596000	-0.376398000
1	1.072477000	-0.135915000	-1.545417000
6	3.550254000	-2.043332000	-0.065679000

1	4.169753000	-1.203616000	0.264417000
1	4.230823000	-2.826667000	-0.413688000
6	0.004294000	-2.203958000	-0.028073000
6	1.864117000	-3.764962000	0.629219000
1	3.347279000	-2.865254000	1.927380000
6	1.787253000	-2.794950000	-1.684293000
1	3.227825000	-1.218785000	-2.040573000
6	0.940472000	-3.323231000	-0.515146000
1	-0.668404000	-1.920608000	-0.836857000
1	-0.614453000	-2.548595000	0.804839000
1	1.270528000	-4.159762000	1.459397000
1	2.523027000	-4.571491000	0.292338000
1	0.338688000	-4.172514000	-0.851508000
1	1.136645000	-2.492164000	-2.511021000
1	2.440914000	-3.585872000	-2.064968000

**2**			
6	4.087621000	1.682862000	2.752174000
6	2.781716000	1.468398000	1.973948000
6	1.691703000	2.449722000	2.439399000
1	2.411740000	0.449203000	2.111859000
1	2.939809000	1.611054000	0.902028000
6	1.447420000	2.218375000	3.940110000
6	3.826040000	1.461189000	4.249571000
6	2.751663000	2.449141000	4.727418000
1	1.092676000	1.196864000	4.096418000
1	0.677308000	2.895221000	4.313605000
6	4.585434000	3.118637000	2.526131000
6	2.210075000	3.885024000	2.219184000
6	3.242448000	3.887873000	4.502399000
1	2.553609000	2.290787000	5.791433000
6	3.513880000	4.108350000	3.005792000
1	4.152984000	4.067772000	5.082178000
1	2.490982000	4.600813000	4.856475000
1	1.466733000	4.614941000	2.538430000
1	2.373656000	4.033478000	1.148363000
1	4.795678000	3.280120000	1.464447000
1	5.521608000	3.284696000	3.068402000
1	3.859583000	5.133168000	2.842104000
1	4.839149000	0.970947000	2.399348000
1	4.748654000	1.605126000	4.820410000
1	3.496253000	0.432600000	4.425496000
6	-0.789275000	2.507534000	1.761967000
6	-1.040821000	3.969681000	2.161396000
6	-2.549455000	4.258800000	2.175541000
6	-3.164387000	4.036384000	0.781506000
6	-2.903025000	2.574091000	0.372178000
1	-2.671991000	4.701050000	0.063957000
6	-4.669708000	4.343817000	0.790289000
6	-1.400430000	2.283311000	0.370040000
1	-0.539698000	4.632384000	1.451690000
1	-0.629023000	4.175814000	3.150992000
6	-3.630556000	1.652625000	1.370605000
1	-3.305050000	2.403930000	-0.632450000
6	-3.277523000	3.337955000	3.173529000

1	-2.706300000	5.300914000	2.473334000
6	-1.511518000	1.581401000	2.756730000
6	-3.017273000	1.875288000	2.763981000
1	-3.509453000	1.210670000	3.481646000
1	-2.875520000	3.507290000	4.178306000
6	-4.778199000	3.637498000	3.173541000
8	0.585293000	2.140235000	1.580427000
1	-1.206385000	1.250717000	0.068855000
1	-0.882610000	2.928849000	-0.344257000
1	-3.473729000	0.610582000	1.072753000
6	-5.135007000	1.952414000	1.376617000
1	-1.322265000	0.544120000	2.467030000
1	-1.114977000	1.715240000	3.762798000
6	-5.389110000	3.416250000	1.781029000
1	-5.656712000	1.300257000	2.082562000
1	-5.542660000	1.751000000	0.386735000
1	-4.966954000	4.671082000	3.475428000
1	-5.301046000	2.994412000	3.886026000
1	-5.067548000	4.213292000	-0.217855000
1	-4.836792000	5.384630000	1.078282000
6	-8.967878000	5.645016000	-0.437617000
6	-7.753133000	5.377543000	0.469732000
6	-7.784915000	3.927080000	0.990290000
1	-7.751869000	6.055694000	1.327497000
1	-6.836997000	5.561491000	-0.091162000
6	-9.098393000	3.719791000	1.763574000
6	-10.266757000	5.430371000	0.352946000
6	-10.315512000	3.983240000	0.866376000
1	-9.099907000	4.388231000	2.628259000
1	-9.115163000	2.696640000	2.147948000
6	-8.928642000	4.675469000	-1.629736000
6	-7.770053000	2.958597000	-0.204657000
6	-10.282499000	3.014259000	-0.324838000
1	-11.230686000	3.826737000	1.444450000
6	-8.983372000	3.228465000	-1.115798000
1	-11.148896000	3.180470000	-0.972565000
1	-10.339786000	1.980530000	0.029892000
1	-7.797731000	1.929503000	0.160665000
1	-6.852579000	3.080184000	-0.783065000
1	-8.015016000	4.833475000	-2.211772000
1	-9.772444000	4.867850000	-2.299451000
1	-8.948832000	2.536596000	-1.962223000
1	-8.923285000	6.675916000	-0.800522000
1	-11.132671000	5.633304000	-0.284963000
1	-10.315328000	6.128697000	1.194018000
8	-6.780826000	3.692053000	1.988383000

**3**			
6	3.925853000	-1.284203000	-0.372392000
6	2.432445000	-1.411459000	-0.704150000
6	1.664589000	-0.137869000	-0.307422000
1	1.992556000	-2.262100000	-0.176985000
1	2.285005000	-1.583363000	-1.773213000
6	1.845381000	0.080953000	1.204715000
6	4.088359000	-1.055737000	1.137897000

6	3.342805000	0.225663000	1.538880000
1	1.425149000	-0.766406000	1.750595000
1	1.316330000	0.977876000	1.529925000
6	4.517732000	-0.091598000	-1.138760000
6	2.277855000	1.051911000	-1.070822000
6	3.930480000	1.421884000	0.774538000
1	3.447752000	0.390226000	2.615096000
6	3.773426000	1.190122000	-0.736456000
1	4.987212000	1.547235000	1.029744000
1	3.418001000	2.343630000	1.067795000
1	1.761434000	1.976416000	-0.813052000
1	2.133877000	0.885897000	-2.141711000
1	4.426783000	-0.255682000	-2.216865000
1	5.584974000	0.007003000	-0.917064000
1	4.185663000	2.042236000	-1.284453000
1	4.440346000	-2.203910000	-0.664970000
1	5.148063000	-0.971728000	1.398041000
1	3.688711000	-1.911061000	1.691511000
6	-0.891293000	0.213224000	-0.315490000
6	-0.839701000	1.747236000	-0.347194000
6	-2.216354000	2.343138000	-0.017659000
6	-3.237684000	1.868039000	-1.057269000
6	-3.311422000	0.338966000	-1.032346000
1	-2.950336000	2.215559000	-2.054732000
1	-4.222346000	2.294222000	-0.840125000
6	-1.935046000	-0.282228000	-1.350915000
1	-0.516449000	2.070768000	-1.339986000
1	-0.107008000	2.117142000	0.372286000
6	-3.762323000	-0.155149000	0.356881000
1	-4.028612000	-0.004851000	-1.785411000
6	-2.650162000	1.871783000	1.375753000
1	-2.147667000	3.434678000	-0.035776000
6	-1.359708000	-0.284817000	1.078398000
6	-2.737484000	0.342353000	1.394342000
1	-3.047684000	0.006993000	2.390031000
1	-1.933587000	2.214824000	2.129242000
1	-3.620388000	2.307064000	1.635106000
8	0.326383000	-0.412343000	-0.746360000
6	-2.035726000	-1.813794000	-1.334180000
1	-1.600310000	0.052821000	-2.337227000
6	-3.851960000	-1.685353000	0.383335000
1	-4.743093000	0.274853000	0.587958000
6	-1.456400000	-1.815414000	1.100468000
1	-0.654574000	0.049849000	1.838917000
6	-2.479032000	-2.288434000	0.056783000
1	-4.595948000	-2.027295000	-0.343547000
1	-4.189624000	-2.021655000	1.369326000
1	-1.756437000	-2.147720000	2.099736000
1	-0.479545000	-2.254692000	0.891766000
1	-1.073255000	-2.252155000	-1.598087000
1	-2.762026000	-2.135048000	-2.087940000
1	-2.541750000	-3.380329000	0.071791000

**CL1**			
6	-0.256946116	0.527051534	-2.180285586

6	1.257657611	0.712195864	-2.128725310
6	1.666158427	1.196730883	-0.742719075
6	-0.930116157	1.863075916	-1.877066433
1	-0.568479133	-0.220909034	-1.447641593
1	-0.558522632	0.171555828	-3.168766509
6	-0.528862515	2.353844273	-0.486027817
6	0.994638482	2.540035673	-0.410261558
1	2.748438553	1.323358221	-0.689430392
1	1.376535810	0.473277611	0.020878880
1	-0.833640152	1.632132888	0.274241339
1	-1.037511826	3.295883392	-0.282537463
6	1.696815503	1.744145006	-3.164189720
1	1.751732203	-0.243586257	-2.338888664
6	1.421298806	3.562382233	-1.466870242
6	1.011675485	3.073929607	-2.858708473
1	1.428930065	1.408643846	-4.169338091
1	2.781953486	1.863831929	-3.122013586
1	2.503075459	3.687895220	-1.427321961
1	0.951197787	4.529756582	-1.280429446
6	-0.504222083	2.892983683	-2.921466806
1	-2.018215421	1.740980147	-1.906110440
1	-1.005508784	3.845250965	-2.733199511
1	-0.795575593	2.556225320	-3.919504090
1	1.319802888	3.822827934	-3.598490121
8	1.437076093	2.788502612	0.927250957
6	1.449613436	4.041579263	1.610238668
6	1.617955811	3.625025689	3.081388658
6	1.712005182	4.845067139	3.990397014
1	2.519792531	3.018080656	3.164603892
1	0.771082512	2.998260518	3.365297471
6	0.169592476	4.875040716	1.499817832
6	0.269920303	6.104267927	2.406012876
1	-0.682218927	4.260272649	1.800355824
1	0.015869273	5.204152490	0.471051604
6	0.434101234	5.670686536	3.860608010
1	-0.427148642	5.075913669	4.173365772
1	0.486842130	6.549959476	4.506572856
6	2.654030981	4.910235696	1.213211808
6	2.915926717	5.690446021	3.580525651
1	1.831216125	4.513326484	5.027993937
6	1.467457515	6.956976105	1.990232843
1	-0.648460811	6.696835889	2.305905112
6	2.745536653	6.130820797	2.128430286
1	2.558617389	5.247401083	0.181852198
1	3.561523358	4.307630600	1.285755550
1	3.833771933	5.108030683	3.687726486
1	2.997011556	6.566205171	4.228339423
1	3.607371367	6.737984039	1.827611022
1	1.349416402	7.289763600	0.955699452
1	1.526142952	7.846012762	2.622158219
6	-1.080847999	-1.591359327	5.234999294
6	-2.194980539	-2.041822950	4.292850177
6	-1.695287889	-3.186113968	3.418146799
6	0.116947692	-1.129351002	4.408308127
1	-0.787025660	-2.417647315	5.885954170

1	-1.434330285	-0.774165562	5.868528808
6	0.625919555	-2.277683731	3.537525658
6	-0.481610583	-2.747299000	2.580721428
1	-2.481705901	-3.517375970	2.737009125
1	-1.401286010	-4.040810852	4.029445134
1	0.935019013	-3.121145058	4.158626994
1	1.493198254	-1.935255953	2.975225792
6	-2.611562551	-0.875042466	3.401447789
1	-3.055987973	-2.383012404	4.879900067
6	-0.913077783	-1.572840583	1.696857239
6	-1.410343773	-0.422374441	2.576182197
1	-2.975977384	-0.048125476	4.015898372
1	-3.420113939	-1.184432847	2.735443533
1	-1.710849199	-1.899828998	1.025903884
1	-0.073140005	-1.219111417	1.095759682
6	-0.294469139	0.037328016	3.511759308
1	0.920186249	-0.806788002	5.081582664
1	0.561802094	0.387975991	2.930827837
1	-0.647747778	0.870183703	4.124917433
1	-1.710148687	0.412038427	1.930136125
8	-0.130804212	-3.950148770	1.896388281
6	0.731754357	-4.084509170	0.766782284
6	1.055599394	-5.588386575	0.779528345
6	1.927183506	-5.970910535	-0.410928285
1	0.115418801	-6.140037273	0.764782325
1	1.561879656	-5.825489500	1.716910467
6	2.052866399	-3.310514797	0.821350885
6	2.925642288	-3.693987427	-0.376888519
1	2.576975308	-3.548069141	1.749635773
1	1.865345736	-2.235544952	0.791026375
6	3.236806337	-5.188000470	-0.342873817
1	3.768064481	-5.436625195	0.578324870
1	3.880882805	-5.454506028	-1.184483495
6	0.013679710	-3.753223359	-0.550829510
6	1.189959555	-5.638532863	-1.706343783
1	2.139979507	-7.045320751	-0.373674852
6	2.198522854	-3.358421418	-1.676939295
1	3.861133457	-3.122387381	-0.327855195
6	0.890085400	-4.142221298	-1.739978090
1	-0.208080586	-2.687820416	-0.607458929
1	-0.929720318	-4.298459910	-0.576775978
1	0.257730711	-6.204897797	-1.760205028
1	1.802632965	-5.915408954	-2.567681561
1	0.361941917	-3.896651268	-2.668203674
1	1.993287654	-2.286736292	-1.719596914
1	2.826129758	-3.614252206	-2.533709725
6	-8.310108359	-1.740431666	0.882619901
6	-6.931501290	-1.174332974	1.214385554
6	-5.863528528	-2.126303210	0.688286133
6	-8.459094972	-3.107613152	1.545829371
1	-8.420911541	-1.840233270	-0.199401520
1	-9.090966000	-1.065351374	1.241261602
6	-7.387136861	-4.066132053	1.020899105
6	-5.994750918	-3.513885268	1.340478825
1	-4.864169770	-1.741047648	0.900353837

1	-5.953429062	-2.236757709	-0.394131169
1	-7.490809994	-4.183678582	-0.059415504
1	-7.525628043	-5.040028174	1.494472896
6	-6.786370035	-1.021210967	2.726343830
1	-6.810668708	-0.195205397	0.735810265
6	-5.865591878	-3.348441303	2.863928720
6	-6.938531319	-2.390601770	3.382358078
1	-7.550262437	-0.340083293	3.109204146
1	-5.809086930	-0.598491011	2.967599131
1	-4.870705841	-2.967372269	3.097700549
1	-5.981964693	-4.308637443	3.364824958
6	-8.319953386	-2.958020650	3.060074157
1	-9.446866768	-3.521009525	1.309816073
1	-8.447875092	-3.928721308	3.545405279
1	-9.096243295	-2.290101245	3.440639293
1	-6.828986260	-2.288880357	4.468622373
8	-4.897616656	-4.226536605	0.766613478
6	-4.673006857	-5.637477935	0.790441022
6	-5.553687012	-6.403368731	-0.210594592
6	-5.100064798	-7.861282585	-0.299935310
1	-5.483293461	-5.919445026	-1.186051812
1	-6.597334204	-6.382120825	0.100229734
6	-4.787368004	-6.305301346	2.164188861
6	-4.335817073	-7.764830938	2.069482858
1	-5.821723770	-6.279718170	2.513013065
1	-4.162798094	-5.769938368	2.882020623
6	-5.223304325	-8.513932429	1.076351413
1	-6.263522621	-8.487315076	1.410318488
1	-4.917680927	-9.561362847	1.021398100
6	-3.212703022	-5.740692138	0.319042037
6	-3.649534517	-7.938764302	-0.768204479
1	-5.743326868	-8.389836746	-1.013580194
6	-2.881687059	-7.836813801	1.608567906
1	-4.427508644	-8.226515938	3.059851751
6	-2.765817064	-7.194308015	0.228761260
1	-2.576451660	-5.191832478	1.015737309
1	-3.142683588	-5.257871297	-0.656659846
1	-3.551230439	-7.489897201	-1.759402420
1	-3.336851077	-8.983205516	-0.840874478
1	-1.723406484	-7.233265243	-0.108304259
1	-2.238444086	-7.308508324	2.315552345
1	-2.555925982	-8.878931361	1.568003016
6	7.124671939	5.004095878	-0.357886944
6	5.916090728	5.083327077	-1.286867246
6	5.374359858	3.680654685	-1.533289341
6	8.197372149	4.133975405	-1.008444456
1	6.827302701	4.575660755	0.601544314
1	7.520194162	6.005130966	-0.171142693
6	7.660041494	2.724019648	-1.252498186
6	6.437445856	2.778685194	-2.181517639
1	4.513478047	3.720502175	-2.203143613
1	5.052968433	3.220581842	-0.598043081
1	7.361507387	2.258032929	-0.312065023
1	8.450132497	2.117740770	-1.693984052
6	6.329231584	5.701291164	-2.620766416

1	5.135152605	5.696013981	-0.822285296
6	6.844714043	3.415778777	-3.514310093
6	7.398298811	4.822034275	-3.266029733
1	6.722463773	6.708096010	-2.463012555
1	5.461649421	5.779431392	-3.280090943
1	5.973493943	3.473982024	-4.170410751
1	7.610355154	2.816992993	-4.010226856
6	8.614693382	4.747237621	-2.344400176
1	9.069218441	4.073447674	-0.346546590
1	9.396903545	4.139351545	-2.805547462
1	9.020867731	5.748724758	-2.185548040
1	7.695962628	5.256846631	-4.227600459
8	5.762895663	1.521867210	-2.264703475
6	6.138864985	0.395187353	-3.059180511
6	5.373920977	-0.761307191	-2.394087280
6	5.600068221	-2.067704072	-3.146721400
1	4.313250140	-0.507954734	-2.371575065
1	5.714127310	-0.848760328	-1.361840055
6	7.628771095	0.039540357	-3.062561746
6	7.850192524	-1.265010517	-3.832122362
1	7.975258033	-0.080290519	-2.033970124
1	8.211860098	0.831227667	-3.535430665
6	7.089828547	-2.400993148	-3.153760683
1	7.449963283	-2.528218542	-2.130904703
1	7.262619232	-3.337982028	-3.688245442
6	5.645690902	0.527703329	-4.509209792
6	5.099294404	-1.916251440	-4.581119210
1	5.049002936	-2.871607015	-2.646718633
6	7.359668542	-1.111666176	-5.270675266
1	8.922450200	-1.493929833	-3.834561681
6	5.867275428	-0.784739446	-5.260836688
1	6.179761335	1.327286501	-5.022137865
1	4.584274428	0.783720055	-4.496481277
1	4.030312672	-1.692053717	-4.579233275
1	5.247381019	-2.848751287	-5.130814244
1	5.511645482	-0.669861231	-6.291419390
1	7.912914685	-0.313732021	-5.771893124
1	7.536157486	-2.037576031	-5.822770189
6	5.717608870	1.887705857	2.310177560
6	5.963647480	1.436826745	3.747993059
6	6.968730777	0.289729962	3.750416969
6	5.179238680	0.711707081	1.499046675
1	6.651585427	2.251971110	1.874902238
1	4.995635635	2.708063741	2.292842013
6	6.188778076	-0.438186276	1.503514214
6	6.441495932	-0.906212391	2.938677398
1	7.167770971	-0.047214471	4.768604931
1	7.918308384	0.610248198	3.318074931
1	7.129455739	-0.108997097	1.056505435
1	5.786152216	-1.259539578	0.907214212
6	4.652909016	0.962098945	4.369302850
1	6.368060535	2.271957202	4.332141473
6	5.110420135	-1.360942002	3.559136594
6	4.108094723	-0.205619003	3.551437565
1	3.926010559	1.777322116	4.386506464

1	4.823852776	0.646015052	5.400865129
1	5.296277570	-1.700490680	4.580258250
1	4.691616695	-2.195202080	2.997088507
6	3.862660204	0.236740057	2.109169605
1	5.013007126	1.023949880	0.461188720
1	3.473221496	-0.601889899	1.526867459
1	3.123259108	1.039254337	2.071131211
1	3.167118168	-0.548574763	3.995442059
8	7.481525471	-1.872019638	3.102115001
6	7.689974214	-3.055571073	2.332893047
6	8.691701804	-3.837160161	3.200808834
6	9.098394438	-5.143174066	2.527932455
1	8.229949787	-4.032539568	4.169967462
1	9.562514797	-3.203199995	3.372941040
6	8.353210482	-2.771394042	0.974710897
6	8.757866620	-4.083981725	0.302984806
1	9.228615708	-2.138787955	1.136146268
1	7.666427981	-2.236490802	0.319467959
6	9.751757230	-4.839653368	1.181526692
1	10.649553616	-4.236187220	1.331653748
1	10.049682616	-5.769520378	0.691347098
6	6.455315810	-3.935823797	2.110429773
6	7.856732618	-6.005513273	2.313901280
1	9.809617614	-5.674671406	3.170132923
6	7.513796245	-4.943645731	0.082999762
1	9.223933181	-3.861126348	-0.664618153
6	6.867857214	-5.245291050	1.433690207
1	5.730779005	-3.423783880	1.474412950
1	5.984013161	-4.148036785	3.072187027
1	7.396009572	-6.240084544	3.276040114
1	8.131578212	-6.947940561	1.834505104
1	5.974050202	-5.860784936	1.278873943
1	6.802559567	-4.419684126	-0.560237493
1	7.788616579	-5.876447508	-0.414526940
6	-4.358972415	-4.859160540	-5.211469751
6	-4.039056892	-4.703451060	-3.726930443
6	-4.959945120	-3.655895074	-3.109423477
6	-4.157412204	-3.515491765	-5.906982976
1	-5.391453565	-5.192217520	-5.337726638
1	-3.705825426	-5.612536817	-5.658618786
6	-5.082685249	-2.463916436	-5.295231481
6	-4.775494483	-2.292113992	-3.797824239
1	-4.749611985	-3.543438614	-2.043958449
1	-6.005034822	-3.953156386	-3.213082694
1	-6.127007552	-2.763294815	-5.405790565
1	-4.944656601	-1.522358347	-5.825386114
6	-2.587848049	-4.261267272	-3.558032446
1	-4.191393742	-5.663358486	-3.219904289
6	-3.313117984	-1.867821633	-3.627296128
6	-2.392406261	-2.917398664	-4.254426312
1	-1.914016987	-5.007085785	-3.987000202
1	-2.349573666	-4.162106247	-2.497677366
1	-3.086788288	-1.769820734	-2.563855028
1	-3.133268782	-0.906187741	-4.111279529
6	-2.706764151	-3.064852543	-5.742021096

1	-4.389202110	-3.617488165	-6.973773143
1	-2.552160471	-2.111918853	-6.254404920
1	-2.034089408	-3.798634461	-6.192243338
1	-1.352600899	-2.590179942	-4.131293237
8	-5.744804013	-1.472788752	-3.142545632
6	-5.806787280	-0.045853511	-3.149357102
6	-7.244997496	0.234109545	-2.680838985
6	-7.497814241	1.733107858	-2.555621290
1	-7.395273704	-0.261601556	-1.721777561
1	-7.937032065	-0.212325680	-3.396999345
6	-5.611311004	0.632650744	-4.509764417
6	-5.859046751	2.138017795	-4.381000475
1	-6.311207425	0.207146939	-5.231580003
1	-4.595371550	0.473512438	-4.875446323
6	-7.290276220	2.395982138	-3.915550750
1	-7.997839885	1.988324278	-4.640921321
1	-7.468661354	3.471204679	-3.840136604
6	-4.838147776	0.576970385	-2.132053543
6	-6.525992462	2.328232678	-1.539635134
1	-8.527601388	1.898357137	-2.219535469
6	-4.882719724	2.739383308	-3.372419302
1	-5.704644016	2.604787396	-5.361362085
6	-5.096294261	2.078365221	-2.012131283
1	-3.807023811	0.414719443	-2.443894417
1	-4.978295096	0.086709920	-1.167145930
1	-6.682365196	1.860300363	-0.564592222
1	-6.702749612	3.400661165	-1.427511862
1	-4.392157300	2.502271224	-1.286773933
1	-3.853298246	2.584409433	-3.706512834
1	-5.050790648	3.816410360	-3.295304128
6	-3.267927840	3.308119540	3.802116283
6	-4.272141281	2.660740254	2.850844549
6	-4.391415770	3.514000527	1.589411145
6	-3.760558019	4.705143218	4.176343662
1	-2.287453768	3.368686831	3.322587575
1	-3.159308993	2.700986970	4.704266601
6	-3.889917032	5.570538567	2.919591793
6	-4.881908770	4.925547160	1.946426163
1	-5.103469321	3.065468242	0.896011803
1	-3.419530785	3.557336157	1.098220078
1	-2.906684543	5.669544391	2.457415448
1	-4.244875523	6.565342786	3.196051147
6	-5.630234042	2.547718172	3.538002778
1	-3.920943395	1.662335230	2.568405181
6	-6.238676483	4.796384009	2.660139091
6	-6.114814740	3.944492882	3.918814745
1	-5.547046386	1.922690521	4.430287767
1	-6.348066444	2.077084265	2.863648345
1	-6.950978671	4.353363125	1.962901204
1	-6.597097828	5.796924792	2.907368030
6	-5.114858677	4.594029136	4.873143446
1	-3.039871114	5.178229051	4.854604104
1	-5.467021125	5.586250839	5.163531630
1	-5.017968233	3.993576145	5.780679953
1	-7.094123306	3.874464332	4.405301205

8	-5.216021068	5.686046673	0.783848197
6	-4.319687226	6.395761011	-0.069198162
6	-3.049759357	5.644761278	-0.479195702
6	-2.267922070	6.468067502	-1.504953764
1	-3.323453688	4.681333769	-0.914874763
1	-2.418789393	5.468413266	0.392032493
6	-3.932994726	7.766794055	0.509762876
6	-3.159108384	8.577695400	-0.529444166
1	-3.313685206	7.645086558	1.398089626
1	-4.844383988	8.291391704	0.803278473
6	-1.887165838	7.821467070	-0.908512611
1	-1.261220362	7.678927669	-0.023979452
1	-1.311222472	8.398639875	-1.635855446
6	-5.165107144	6.628176073	-1.333050127
6	-3.118463318	6.681498740	-2.754478271
1	-1.355008905	5.922611101	-1.776093693
6	-4.016566437	8.793693813	-1.774317664
1	-2.891220959	9.548981355	-0.097438292
6	-4.388592485	7.436340350	-2.367395998
1	-6.080218968	7.147149179	-1.045239968
1	-5.450533181	5.656463413	-1.739624528
1	-3.375895243	5.717055259	-3.198315872
1	-2.554712763	7.252269962	-3.496133177
1	-5.013697134	7.582287754	-3.255654437
1	-4.921592467	9.345010171	-1.511049216
1	-3.464461008	9.384895368	-2.508747876
**CL2**			
6	-9.113359698	-1.126924511	0.967899038
6	-7.667781496	-0.694669206	0.750028391
6	-6.729747333	-1.374312416	1.761855655
1	-7.573270731	0.386698501	0.860774026
1	-7.335604004	-0.955894763	-0.254359348
6	-7.184578048	-0.978441325	3.169927588
6	-9.549348452	-0.735366471	2.377922444
6	-8.633801437	-1.420870549	3.389487506
1	-7.110070566	0.105042161	3.282847157
1	-6.553632238	-1.450505032	3.924817811
6	-9.222140991	-2.640170180	0.796232876
6	-6.862694345	-2.896648726	1.596862664
6	-8.744205911	-2.936060268	3.228311720
1	-8.936717940	-1.139367479	4.404914165
6	-8.313664432	-3.324368173	1.814755839
1	-9.774135220	-3.253524033	3.405576986
1	-8.109123031	-3.437669651	3.962503543
1	-6.228379931	-3.415814826	2.314468569
1	-6.530607859	-3.171279214	0.593388296
1	-8.924206166	-2.921733888	-0.216125466
1	-10.255691128	-2.961004301	0.944582288
1	-8.386204090	-4.411745560	1.696437515
1	-9.753617497	-0.626486181	0.232693447
1	-10.584741348	-1.038220805	2.548979700
1	-9.492401937	0.348837797	2.497786815
6	-4.239949493	-0.935811641	2.133183195
6	-3.930247407	-2.251098439	2.854235672
6	-2.543959050	-2.177018032	3.499704747

6	-1.462801630	-1.954516725	2.442351073
6	-1.767414428	-0.647125663	1.710214214
1	-1.486963420	-2.785358765	1.726073477
6	-0.073180232	-1.901419308	3.080378239
6	-3.149343883	-0.715343913	1.072369536
1	-3.958090738	-3.073999267	2.136630482
1	-4.669389033	-2.445380399	3.633000302
6	-1.705255743	0.503173069	2.715823877
1	-1.017340104	-0.487439722	0.924998851
6	-2.479065750	-1.028262298	4.506970983
1	-2.350347167	-3.123137123	4.018889319
6	-4.168385043	0.219534342	3.142912536
6	-2.780547242	0.281276790	3.777214586
1	-2.754956652	1.109260997	4.495337282
1	-3.228119551	-1.188198419	5.291564030
6	-1.096393556	-0.969939316	5.144264135
8	-5.440852140	-0.902175247	1.362241080
1	-3.372227970	0.207465234	0.535011043
1	-3.199199300	-1.537657858	0.355759363
1	-1.901818323	1.449608903	2.196622281
6	-0.318023111	0.570229529	3.350967742
1	-4.393746806	1.151778794	2.621180935
1	-4.910200224	0.086811981	3.929317331
6	-0.005811122	-0.745169341	4.083099086
1	-0.267294948	1.392482233	4.067978966
1	0.415387586	0.756906125	2.568446258
1	-0.878867615	-1.899400647	5.672909168
1	-1.041970590	-0.157710002	5.870421799
1	0.671071920	-1.770501317	2.293507609
1	0.133291907	-2.841023330	3.596612315
6	4.512833659	-2.193318614	3.917562741
6	3.008202065	-2.177300589	4.190123240
6	2.530364942	-0.732914983	4.404909015
1	2.775002972	-2.761408288	5.082802943
1	2.490847292	-2.629237534	3.344197547
6	3.316411679	-0.129978030	5.581665114
6	5.270517307	-1.592132805	5.099294477
6	4.815310505	-0.148357188	5.301568920
1	3.084762988	-0.703378356	6.480157348
1	2.968402531	0.891724580	5.743846132
6	4.809751299	-1.379791197	2.659131716
6	2.843248797	0.086474347	3.151834848
6	5.106354978	0.668566901	4.043354233
1	5.347148239	0.289015236	6.154395374
6	4.345956729	0.060132857	2.866893381
1	6.179402051	0.666019972	3.835939850
1	4.796931405	1.705680500	4.194498793
1	2.519455630	1.117469697	3.309616952
1	2.315381433	-0.321467763	2.288722526
1	4.305998096	-1.825537551	1.798769471
1	5.885172777	-1.390014645	2.462885583
1	4.536578783	0.644221331	1.958287234
1	4.834343174	-3.230136296	3.765089809
1	6.345689570	-1.622968407	4.906589513
1	5.075214862	-2.172389383	6.003724718

8	1.178936524	-0.671147876	4.870248334
6	6.446170896	-3.991636823	0.296454112
1	6.080583303	-5.018681709	0.347507483
1	6.172487021	-3.498015070	1.228355946
6	-5.636143728	-2.054367862	-4.970547191
6	-4.189778151	-2.513623428	-5.116403147
6	-3.333775766	-2.023677154	-3.935599643
1	-3.753374843	-2.128934632	-6.039067890
1	-4.134445710	-3.602762663	-5.154868570
6	-3.398436679	-0.488626923	-3.895302516
6	-5.679513862	-0.528188997	-4.935816469
6	-4.849972117	-0.031715016	-3.754044522
1	-2.959832274	-0.093711707	-4.814089724
1	-2.824225866	-0.103230599	-3.053378974
6	-6.215571410	-2.618757079	-3.674702245
6	-3.932493538	-2.584299461	-2.642945139
6	-5.432205698	-0.590186832	-2.457287851
1	-4.875477554	1.064528952	-3.728086468
6	-5.382195932	-2.115634321	-2.498802853
1	-6.466544117	-0.257046782	-2.344968531
1	-4.871414765	-0.227585290	-1.594247814
1	-3.362294503	-2.237659006	-1.779599153
1	-3.895528992	-3.675515708	-2.668877535
1	-6.201212210	-3.710685330	-3.705785050
1	-7.254146344	-2.299752173	-3.558070593
1	-5.786565670	-2.514194536	-1.561173372
1	-6.219146416	-2.419478932	-5.823604702
1	-6.711882689	-0.184487929	-4.835824346
1	-5.279856735	-0.124506642	-5.868625305
6	-0.893081847	-2.605371403	-3.434896524
6	-0.923208634	-3.855912525	-2.541328981
6	0.424172959	-4.036123142	-1.841711999
6	1.551024611	-4.194744176	-2.860645267
6	1.587023550	-2.949113141	-3.747287116
1	1.353654963	-5.074936711	-3.483509330
6	2.897138015	-4.375923793	-2.161874238
6	0.249410367	-2.768304377	-4.451018264
1	-1.149063253	-4.725800560	-3.161435030
1	-1.705316506	-3.766706167	-1.787323922
6	1.887617382	-1.729874885	-2.874305196
1	2.375851354	-3.061515505	-4.500603934
6	0.730710831	-2.818627671	-0.969421260
1	0.379355944	-4.931567574	-1.210596257
6	-0.587249725	-1.384332281	-2.562853470
6	0.757220836	-1.574979991	-1.857220478
1	0.951712009	-0.695688139	-1.231501655
1	-0.053766796	-2.707631498	-0.211707753
6	2.075841004	-2.984923825	-0.271717088
8	-2.035981437	-2.508245433	-4.281035478
1	0.269581758	-1.886025531	-5.093176342
1	0.026214434	-3.629745278	-5.081837888
1	1.928638938	-0.834607751	-3.507072243
6	3.238185045	-1.896703666	-2.175664985
1	-0.552131073	-0.491210814	-3.190100719
1	-1.364589508	-1.247686860	-1.808527622

6	3.214005571	-3.148295995	-1.293161910
1	3.453352602	-1.020864068	-1.558775544
1	4.016380590	-1.982047079	-2.936199380
1	2.067780580	-3.859799604	0.380297384
1	2.292928752	-2.110943273	0.346313520
1	3.666155871	-4.519626848	-2.919224136
1	2.880879942	-5.261904991	-1.523525826
6	7.610513252	-3.978615449	-2.363068740
6	6.092454528	-4.001112828	-2.162878371
6	5.729498613	-3.277168863	-0.862157725
1	5.744880818	-5.034901010	-2.114334300
1	5.618452577	-3.513545368	-3.016718266
6	8.303943530	-4.689957980	-1.203547410
6	7.957622265	-3.971912601	0.098842767
6	8.102419797	-2.534366734	-2.437721227
6	6.241526347	-1.830197331	-0.938396386
6	8.442593343	-2.525415620	0.034217316
1	8.438583316	-4.484054120	0.939856436
6	7.757227675	-1.819349556	-1.132680769
1	9.526679080	-2.499545901	-0.099038319
1	8.206707287	-2.011632099	0.968574969
1	5.969653347	-1.308511643	-0.020746187
1	5.774370693	-1.305286211	-1.770813613
1	7.631091490	-2.021454622	-3.279663959
1	9.182630592	-2.518405207	-2.599622854
1	8.101670456	-0.780369336	-1.183201986
1	7.848179724	-4.494153101	-3.301241876
1	9.385201823	-4.686426983	-1.359222911
1	7.975468646	-5.730423765	-1.153546998
8	4.357271327	-3.343813637	-0.463941472
6	5.920102761	3.892282818	0.906753459
1	5.717602097	3.280285915	1.788312114
1	5.256235970	4.755550854	0.933792659
6	-5.564841276	4.959862740	0.669156146
6	-4.136894316	5.177398288	0.169399086
6	-3.873058455	4.310903677	-1.072983852
1	-3.437856062	4.923729730	0.966215249
1	-3.979608767	6.224626856	-0.096610501
6	-4.083865702	2.836440787	-0.717660931
6	-5.745575310	3.488853059	1.038887068
6	-5.507155142	2.622643302	-0.196758304
1	-3.376161374	2.521596013	0.050736390
1	-3.925775558	2.223669428	-1.607094724
6	-6.566465800	5.342027944	-0.417767059
6	-4.901955037	4.688776328	-2.151999456
6	-6.514934132	2.999639694	-1.279628308
1	-5.627690430	1.566162135	0.069061732
6	-6.325138129	4.470021279	-1.647982283
1	-7.531774745	2.832556442	-0.916829682
1	-6.366629704	2.372370514	-2.160453580
1	-4.712110922	4.082956698	-3.039690738
1	-4.745872638	5.732982783	-2.425819824
1	-6.448034100	6.395821089	-0.678795364
1	-7.585823388	5.198898663	-0.051502803
1	-7.033050579	4.745479244	-2.437872154

1	-5.731821326	5.583566439	1.555379349
1	-6.755268727	3.319975427	1.420655235
1	-5.041319544	3.218620724	1.829958867
6	-1.336862921	4.160047903	-1.319559823
6	-1.050217149	2.774008529	-1.918938655
6	0.411057816	2.394816059	-1.692234867
6	1.356103084	3.403220079	-2.342745842
6	1.075013302	4.782948875	-1.746858920
1	1.167640655	3.430752755	-3.422284775
6	2.815039306	3.010351324	-2.105811033
6	-0.382118271	5.169547028	-1.980016190
1	-1.280622855	2.801480987	-2.985960879
1	-1.685013586	2.018492083	-1.456089713
6	1.376613060	4.738222761	-0.247969962
1	1.722083695	5.526837974	-2.226721494
6	0.701622324	2.351925030	-0.192681676
1	0.592388961	1.405583590	-2.125681832
6	-1.027188664	4.140918576	0.180357082
6	0.428740840	3.732665520	0.404577586
1	0.620340647	3.701868141	1.484197575
1	0.043209508	1.614789491	0.282171254
6	2.151856031	1.963464621	0.053912272
8	-2.626385315	4.642296788	-1.689890673
1	-0.584962997	6.159921092	-1.568749976
1	-0.602959423	5.204630670	-3.047657100
1	1.202984643	5.730812920	0.184715238
6	2.835099296	4.347487366	0.002105562
1	-1.197489876	5.135602834	0.597519111
1	-1.673264689	3.433520574	0.701737071
6	3.113763399	2.967204460	-0.598978396
1	3.026290882	4.321082509	1.076941726
1	3.483958427	5.100766903	-0.447864727
1	2.365275102	0.972797233	-0.351292615
1	2.363764870	1.937288797	1.122737705
1	3.462652244	3.732396874	-2.602794818
1	3.017426906	2.025032380	-2.531741408
6	8.084507665	2.354830903	-0.336448258
6	6.641159330	1.865272899	-0.348893065
6	5.657931081	3.048033283	-0.350511695
1	6.438263975	1.248912912	0.527728976
1	6.449148128	1.255761878	-1.232840533
6	8.324982554	3.190857965	0.918208328
6	7.368623637	4.380731832	0.915651890
6	8.339270662	3.204496228	-1.579307608
6	5.931051597	3.895918494	-1.596414689
6	7.619159658	5.236015954	-0.325529171
1	7.535227792	4.984954486	1.814977008
6	7.379025755	4.391998892	-1.576261273
1	8.646031757	5.608285084	-0.321225397
1	6.950715929	6.100394543	-0.323930873
1	5.261229348	4.756936220	-1.628294463
1	5.759764602	3.291660018	-2.489623271
1	8.186958385	2.603396348	-2.478575862
1	9.371494212	3.561695826	-1.585009398
1	7.553990137	5.004592325	-2.468582340

1	8.759856580	1.491474527	-0.335955212
1	9.358583940	3.543527244	0.941497974
1	8.157051850	2.581125330	1.808564595
8	4.389059696	2.394385965	-0.310698810
**CL3**			
6	-2.946193211	4.620269430	2.491083594
6	-3.384341798	3.459737620	3.376681678
6	-3.719951966	2.215832312	2.534535501
1	-4.266747245	3.729304422	3.958716638
1	-2.592279901	3.201952345	4.081289333
6	-4.842815787	2.593062093	1.553612364
6	-4.073461724	4.977183089	1.524884425
6	-4.397884167	3.756490894	0.667521004
1	-5.731880090	2.866532883	2.125566254
1	-5.097766073	1.744750861	0.920378431
6	-1.705884829	4.211915516	1.700598940
6	-2.469890424	1.824808753	1.739809015
6	-3.159621684	3.339830575	-0.125029128
1	-5.210280300	4.005872785	-0.026463984
6	-2.035296205	2.990080957	0.847744731
1	-2.844233374	4.152148543	-0.784427092
1	-3.391902165	2.474714372	-0.750243412
1	-2.671308660	0.954323664	1.112200783
1	-1.663199431	1.573074161	2.429721188
1	-0.884365505	3.984947144	2.382999671
1	-1.390136384	5.035501928	1.055749861
1	-1.143357982	2.688486724	0.284357619
1	-2.712305269	5.486530074	3.120257035
1	-3.771141456	5.811407700	0.887122184
1	-4.959591978	5.285584336	2.083588610
6	-4.281801521	-0.132686311	3.389182685
6	-5.014303464	-0.577470620	2.118895837
6	-5.251308912	-2.086059630	2.137086067
6	-3.909492760	-2.807186679	2.221591712
6	-3.193417053	-2.387857882	3.502705589
1	-3.296499564	-2.557337367	1.351600874
1	-4.068999934	-3.888133225	2.220896796
6	-2.936811605	-0.877900864	3.505656194
1	-4.423671776	-0.324745944	1.235934557
1	-5.969799056	-0.052959398	2.063077886
6	-4.036649617	-2.761507345	4.721788116
1	-2.225947699	-2.902583381	3.562751980
6	-6.107573478	-2.459906358	3.342802475
1	-5.765461061	-2.377324484	1.213213774
6	-5.131901657	-0.530578528	4.616592148
6	-5.381307647	-2.039478037	4.616846840
1	-5.997807624	-2.294153996	5.486873298
1	-7.076112905	-1.957588387	3.287114567
1	-6.287771454	-3.537583392	3.352519293
8	-4.151640735	1.283431080	3.524851699
6	-2.227331878	-0.469869517	4.794591007
1	-2.300308040	-0.634612574	2.649627917
6	-3.315363163	-2.357547700	6.005640254
1	-4.205885803	-3.845295052	4.725331038
6	-4.421534898	-0.123644137	5.906768558

1	-6.085750915	0.003481414	4.550254286
6	-3.081881173	-0.848390332	6.000423164
1	-2.359970733	-2.884537640	6.076051224
1	-3.916783022	-2.638745367	6.873230365
1	-5.047481812	-0.378974352	6.765107071
1	-4.265108426	0.955083407	5.903980978
1	-2.068027969	0.610283586	4.787144344
1	-1.254813283	-0.967358911	4.854817191
1	-2.566268745	-0.552240577	6.921296549
6	4.512564439	6.438650834	0.732579142
6	4.475324490	5.983629962	-0.722137633
6	3.036923825	5.663514361	-1.167293048
1	5.084281567	5.090024780	-0.857770780
1	4.877386540	6.760903449	-1.374700013
6	2.484019932	4.562313762	-0.248100086
6	3.961420146	5.328337414	1.624535173
6	2.525067203	5.024644796	1.207963771
1	3.089310738	3.663284754	-0.383183935
1	1.455015623	4.319553367	-0.513529969
6	3.663139524	7.698006149	0.886273592
6	2.192258690	6.929561751	-0.995060164
6	1.672303893	6.281410391	1.365811920
1	2.118190435	4.229090341	1.843249381
6	2.229203037	7.383146102	0.467445950
1	1.682426701	6.609820266	2.407164300
1	0.637163375	6.065646900	1.093702499
1	1.155435844	6.738943097	-1.279069907
1	2.586112900	7.722967449	-1.632991751
1	4.068162045	8.498812898	0.263534534
1	3.679698195	8.038546298	1.923992662
1	1.613043674	8.284314250	0.570141199
1	5.548355845	6.657047180	1.015465848
1	3.987641851	5.639411436	2.671628855
1	4.577476161	4.431700203	1.523420994
6	2.243621913	5.100497899	-3.539151967
6	0.958264033	4.380260180	-3.113653858
6	0.041799108	4.165132037	-4.317128552
6	-0.317835611	5.518297673	-4.925112598
6	0.959243428	6.222477263	-5.377612789
1	-0.841352727	6.132471026	-4.187841265
1	-0.986100843	5.377956164	-5.778186300
6	1.894525039	6.456002239	-4.186066440
1	0.426807280	4.971418683	-2.365567439
1	1.221231867	3.418078135	-2.667477926
6	1.679750345	5.379374011	-6.430222861
1	0.700050120	7.195825269	-5.811835455
6	0.752515225	3.310500925	-5.362544154
1	-0.871230630	3.657043306	-3.983932478
6	2.954051835	4.253047919	-4.617397929
6	2.023968521	4.025087627	-5.808839033
1	2.545997132	3.407922249	-6.549918624
1	1.005469001	2.333986148	-4.943566789
1	0.094454277	3.146047296	-6.219070257
8	3.223885619	5.197810217	-2.505596639
6	3.169825954	7.157551963	-4.649961889

1	1.374655441	7.090262092	-3.463293281
6	2.949715963	6.088538325	-6.894244529
1	1.014357988	5.226117005	-7.288526218
6	4.230795574	4.951324103	-5.082435507
1	3.214102352	3.290107745	-4.164578572
6	3.875562835	6.301529744	-5.698676104
1	2.693164029	7.050391856	-7.345172477
1	3.454018321	5.486230459	-7.653870813
1	4.743394994	4.326358355	-5.818374320
1	4.894143561	5.093582063	-4.229259034
1	3.833014584	7.306141342	-3.796091902
1	2.921061620	8.136881323	-5.066918859
1	4.790187557	6.807080498	-6.028339910
6	-5.202139049	-5.742090328	-3.350055769
6	-3.744509535	-5.324751622	-3.197131576
6	-3.627541883	-3.818034657	-2.905468911
1	-3.187362196	-5.546558960	-4.109438878
1	-3.269632909	-5.869520270	-2.380222919
6	-4.271552716	-3.050828893	-4.064029359
6	-5.825539810	-4.967329388	-4.509036006
6	-5.736226032	-3.471766966	-4.213707504
1	-3.732796797	-3.261186538	-4.990220186
1	-4.230217040	-1.975759232	-3.876829540
6	-5.954064812	-5.435340117	-2.056968121
6	-4.407533949	-3.514979947	-1.615163388
6	-6.501832400	-3.165106197	-2.928450831
1	-6.179822309	-2.911103938	-5.045413654
6	-5.868302380	-3.937799288	-1.773108475
1	-7.546700101	-3.460643089	-3.043624093
1	-6.487785965	-2.092106612	-2.725373244
1	-4.372482231	-2.449652417	-1.387027828
1	-3.941499407	-4.052809364	-0.787471936
1	-5.514821847	-5.997884539	-1.230001266
1	-6.999876762	-5.736443141	-2.150828572
1	-6.406207664	-3.713420532	-0.843706769
1	-5.250333111	-6.817047859	-3.557656349
1	-6.870914438	-5.257273065	-4.636078707
1	-5.297958412	-5.198653285	-5.437320798
6	-1.463272654	-2.445136316	-2.790329005
6	-2.028492035	-1.324759121	-1.909864499
6	-1.067893820	-0.138542069	-1.875377879
6	0.275620620	-0.577999169	-1.302169926
6	0.859549917	-1.674671719	-2.187219150
1	0.142535439	-0.956467883	-0.285820209
1	0.959158082	0.273449006	-1.258091960
6	-0.086223727	-2.873700375	-2.236315824
1	-2.181760295	-1.711684698	-0.899357561
1	-2.990535849	-0.990276266	-2.302501465
6	1.072211896	-1.157521129	-3.610296336
1	1.820731016	-2.001865609	-1.773778433
6	-0.865097823	0.391684015	-3.291288694
1	-1.495568665	0.651103130	-1.245826374
6	-1.229985080	-1.915166013	-4.220319414
6	-0.280305489	-0.713138083	-4.166123737
1	-0.144458379	-0.331373628	-5.185548191

1	-1.815501179	0.727941658	-3.710135025
1	-0.187875579	1.247781420	-3.268445746
8	-2.217661171	-3.654905876	-2.741312257
6	0.508835850	-3.975381928	-3.111480305
1	-0.240330449	-3.260920400	-1.222571860
6	1.670760194	-2.253455140	-4.488205083
1	1.753053746	-0.298840481	-3.586901531
6	-0.630145352	-3.012543549	-5.097459374
1	-2.177810848	-1.583450069	-4.652773978
6	0.717736600	-3.446276440	-4.527119896
1	2.639884480	-2.561189510	-4.087015526
1	1.832658829	-1.870905130	-5.499126403
1	-0.507174347	-2.641941346	-6.118466539
1	-1.306144019	-3.868919720	-5.122167896
1	-0.170144095	-4.828391410	-3.125675018
1	1.465610541	-4.299680733	-2.694362036
1	1.144004516	-4.236382097	-5.155737411
6	5.081706156	0.158606504	-3.487149409
6	6.094873274	-0.849353262	-2.955763072
6	7.043365786	-0.197387213	-1.933674563
1	6.690213614	-1.257092409	-3.775009320
1	5.586855434	-1.682040016	-2.469364980
6	7.768545120	0.961659250	-2.624824485
6	5.826428037	1.302552923	-4.170908242
6	6.748074751	1.972850847	-3.155235895
1	8.366238319	0.577153906	-3.453526724
1	8.433969466	1.467874483	-1.923126892
6	4.251174829	0.701924158	-2.327080738
6	6.191262476	0.360124106	-0.781368805
6	5.916713391	2.523874725	-1.999039471
1	7.287152288	2.794764917	-3.640506340
6	5.179555081	1.372103573	-1.319269157
1	5.198221905	3.256977297	-2.372766992
1	6.569655286	3.024445830	-1.278842683
1	6.821897466	0.850945265	-0.040913887
1	5.677245088	-0.468413032	-0.290177134
1	3.704265982	-0.112810593	-1.847002582
1	3.521461679	1.428398786	-2.693934071
1	4.589110125	1.759391751	-0.481096519
1	4.424246089	-0.338634798	-4.209877954
1	5.117817471	2.033298748	-4.564458232
1	6.411357215	0.916540330	-5.008960851
6	9.123787768	-1.241743580	-0.862783675
6	9.139822325	-0.325137494	0.365939482
6	10.467176965	-0.450389014	1.110988149
6	10.660782388	-1.889439858	1.578852178
6	10.669234090	-2.810821911	0.362657308
1	9.851134733	-2.173616915	2.255021469
1	11.603123876	-1.981470069	2.124081368
6	9.349742876	-2.698619559	-0.401029775
1	8.314665713	-0.602632792	1.025507559
1	9.004796165	0.713544364	0.059101530
6	11.816189777	-2.435718266	-0.576980447
1	10.796400638	-3.848409220	0.693863715
6	11.609241594	-0.057008428	0.177871263

1	10.453331563	0.219902135	1.978520248
6	10.293286487	-0.878072647	-1.800521911
6	11.613785805	-0.989846620	-1.030622767
1	12.432686502	-0.705532527	-1.702961801
1	11.484454098	0.978145180	-0.150162642
1	12.563569979	-0.127773631	0.705116617
8	7.858011489	-1.298482814	-1.523094123
6	9.367256336	-3.636882212	-1.606816173
1	8.520975316	-2.976196787	0.258215404
6	11.838801377	-3.367339727	-1.786516014
1	12.768089369	-2.516007737	-0.038207837
6	10.310937313	-1.815020965	-3.007027500
1	10.186883613	0.153549436	-2.145396040
6	10.514174112	-3.253072922	-2.537222182
1	11.993367105	-4.398064909	-1.458048749
1	12.667537430	-3.097850248	-2.446110920
1	11.113761069	-1.522966821	-3.689023996
1	9.361957849	-1.737465776	-3.540517596
1	8.418080160	-3.555430227	-2.137056850
1	9.490841095	-4.667893076	-1.265324383
1	10.524796989	-3.923638904	-3.404209827
6	-5.875794172	1.405225474	-5.605701915
6	-6.865103346	0.594597921	-4.775718376
6	-6.977092097	1.152213047	-3.345078956
1	-6.552913750	-0.448827951	-4.715202497
1	-7.854617297	0.615268736	-5.235728359
6	-5.580077388	1.110162842	-2.703300971
6	-4.497578715	1.346863322	-4.951705886
6	-4.591417443	1.921086862	-3.540346314
1	-5.258281872	0.068236404	-2.632736988
1	-5.610822949	1.520316681	-1.694126191
6	-6.349329485	2.855160536	-5.679793781
6	-7.438042026	2.610461515	-3.435479762
6	-5.060574712	3.372782343	-3.609712012
1	-3.605144533	1.883771702	-3.062670333
6	-6.439146441	3.422409353	-4.264798602
1	-4.350272453	3.966136014	-4.190196557
1	-5.106703247	3.797366835	-2.604240859
1	-7.510010267	3.050912252	-2.439315466
1	-8.422125455	2.653723975	-3.905586764
1	-7.326877494	2.903097371	-6.164345012
1	-5.650625885	3.448422945	-6.274070657
1	-6.784450058	4.461950727	-4.310101046
1	-5.821365706	0.983476636	-6.615595204
1	-3.777018697	1.920558037	-5.539817479
1	-4.149947018	0.311503006	-4.914728857
6	-8.642280785	0.434445266	-1.534758859
6	-7.816569037	0.953274621	-0.352639014
6	-8.660155550	0.977522033	0.920134577
6	-9.866009055	1.888925044	0.709530627
6	-10.705754881	1.352630084	-0.447383577
1	-9.531652470	2.905821172	0.488220275
1	-10.469011265	1.926109883	1.619882285
6	-9.883147333	1.326001873	-1.740311923
1	-7.457592227	1.963579616	-0.557200618

1	-6.953857310	0.297755494	-0.214580816
6	-11.196956911	-0.059994723	-0.129767459
1	-11.571230563	2.009104342	-0.599681149
6	-9.135884276	-0.434475275	1.246874258
1	-8.049741222	1.362364985	1.746078980
6	-9.151559677	-0.984932836	-1.199586042
6	-9.977405843	-0.956902617	0.086688680
1	-10.312315596	-1.976414732	0.311950419
1	-8.276873882	-1.091079409	1.402769788
1	-9.727411283	-0.426887955	2.165679655
8	-7.879479385	0.235418514	-2.726244509
6	-10.731649743	0.786521072	-2.890370692
1	-9.573121692	2.349315874	-1.967955950
6	-12.049039744	-0.591808673	-1.279703544
1	-11.797738995	-0.036294282	0.787488054
6	-9.998968611	-1.525521927	-2.349878055
1	-8.279326132	-1.631977986	-1.062884063
6	-11.212430848	-0.623478241	-2.556807354
1	-12.922974185	0.049196035	-1.420313916
1	-12.405625288	-1.597146389	-1.042555546
1	-10.321947379	-2.543557382	-2.118051945
1	-9.394544205	-1.551020510	-3.256358674
1	-10.132287588	0.759859900	-3.802074314
1	-11.585971089	1.447170645	-3.059452657
1	-11.820190806	-1.007786234	-3.383856621
6	3.821512516	0.722506371	2.525541202
6	3.597303405	1.706329923	3.668096326
6	2.312760254	1.367545708	4.445661503
1	3.515300360	2.724662759	3.284413066
1	4.435552070	1.684448275	4.365841979
6	1.133373174	1.412453323	3.470538042
6	2.634443236	0.780043416	1.567030993
6	1.361374095	0.420849320	2.328226194
1	1.032489133	2.420720687	3.063012264
1	0.208688338	1.150661664	3.986956648
6	3.953573078	-0.689477235	3.090318826
6	2.450806176	-0.062786331	4.994965362
6	1.488162632	-0.992955925	2.889458800
1	0.504500437	0.470412680	1.646080857
6	2.677248365	-1.047664057	3.846778268
1	1.631283955	-1.705110225	2.073236774
1	0.571254777	-1.269198718	3.415168950
1	1.552532282	-0.354390115	5.538052876
1	3.290171013	-0.091096875	5.692579344
1	4.814159520	-0.745478233	3.760243088
1	4.112880641	-1.400573995	2.276376625
1	2.766935558	-2.059578151	4.259198461
1	4.739881519	0.991202852	1.991582816
1	2.783994981	0.078246274	0.743442064
1	2.541954368	1.782470325	1.143764210
6	1.215963004	2.753171506	6.301717182
6	0.469918074	1.584590093	6.955548306
6	-0.567703643	2.100549283	7.950733963
6	0.125710362	2.905602054	9.045411206
6	0.854881934	4.085461221	8.409089422

1	0.837976365	2.272731592	9.579933907
1	-0.610948671	3.266723888	9.767138414
6	1.897275840	3.592127679	7.405283647
1	1.191834011	0.945834291	7.468944685
1	-0.038038983	0.991263088	6.194484758
6	-0.140482897	4.990361422	7.680220920
1	1.363534978	4.662337472	9.190679264
6	-1.572258203	2.988271941	7.221400008
1	-1.089181824	1.245543843	8.397005103
6	0.209717803	3.677129897	5.586144971
6	-0.834758673	4.168364817	6.593858619
1	-1.555431343	4.804854035	6.065000397
1	-2.088879206	2.412739071	6.449573414
1	-2.326436221	3.352773350	7.922801879
8	2.310804848	2.355779239	5.475377934
6	2.627938517	4.783083521	6.786153610
1	2.626441974	2.950551678	7.911066717
6	0.582230825	6.182235023	7.056694214
1	-0.890437451	5.352943159	8.393588954
6	0.938446941	4.867275202	4.965681582
1	-0.308804926	3.121006572	4.800525329
6	1.624834057	5.677104256	6.062144728
1	1.067392975	6.772207713	7.838063566
1	-0.138874055	6.827555226	6.548199041
1	0.225319320	5.492812183	4.421472704
1	1.688645491	4.505877612	4.260532078
1	3.376190963	4.415333345	6.084146804
1	3.136764298	5.347798725	7.571207014
1	2.148247523	6.530270746	5.614370224
6	5.864884143	-4.891986847	0.763552993
6	4.700678948	-5.519337885	1.524335335
6	3.465555086	-5.674397493	0.617928603
1	4.427648363	-4.900868322	2.381862060
1	4.974648831	-6.505094439	1.902848977
6	3.070278086	-4.283505032	0.114434478
6	5.454074972	-3.512225610	0.255209455
6	4.238089937	-3.661295858	-0.655222825
1	2.810762216	-3.649176940	0.965484350
1	2.201047971	-4.348532887	-0.541445963
6	6.238172831	-5.783422284	-0.417694756
6	3.858638718	-6.553450007	-0.581135800
6	4.611987488	-4.543058350	-1.845052651
1	3.931004047	-2.670845407	-1.016503750
6	5.027783908	-5.922167182	-1.336891874
1	5.436781425	-4.087455955	-2.397838275
1	3.763497742	-4.637406085	-2.528122123
1	3.018760594	-6.667218641	-1.265380938
1	4.131938177	-7.544482999	-0.213132061
1	6.548087858	-6.767438661	-0.059057265
1	7.072877149	-5.343849556	-0.966102146
1	5.287259914	-6.561348673	-2.188979962
1	6.724483037	-4.795128965	1.436802200
1	6.274120068	-3.049733256	-0.298864941
1	5.212129642	-2.865737102	1.102768834
6	1.117883446	-6.448059880	1.298694016

6	0.698397041	-6.910597433	-0.101468739
6	-0.802626604	-7.192310901	-0.148524629
6	-1.152357053	-8.289773607	0.851924794
6	-0.764503551	-7.825747201	2.252998001
1	-0.615784480	-9.208351722	0.603195310
1	-2.223161396	-8.505269565	0.811993121
6	0.735505714	-7.539342276	2.323353006
1	1.251139030	-7.816897578	-0.357293442
1	0.931366248	-6.138753924	-0.836543983
6	-1.525239725	-6.549927667	2.619687512
1	-1.007630365	-8.613069880	2.976737999
6	-1.570234605	-5.921323018	0.201508981
1	-1.070521663	-7.516678508	-1.161069721
6	0.334183330	-5.175118003	1.675625233
6	-1.168365116	-5.465687047	1.601709950
1	-1.709261959	-4.546117422	1.850748000
1	-1.361435559	-5.141115568	-0.533766874
1	-2.645212611	-6.121230306	0.177997392
8	2.528698913	-6.316390800	1.481356172
6	1.113474333	-7.091927934	3.734898473
1	1.298808241	-8.444638405	2.073681860
6	-1.152529165	-6.096811312	4.029111608
1	-2.603979927	-6.745840976	2.575281151
6	0.708695821	-4.726533677	3.087098459
1	0.566216864	-4.374052551	0.967637150
6	0.347888542	-5.820034319	4.088881678
1	-1.417392048	-6.873410005	4.750925830
1	-1.712931078	-5.194900634	4.288793282
1	0.180358948	-3.801395795	3.332083469
1	1.781099100	-4.530020390	3.132690815
1	2.186422126	-6.905359615	3.773868304
1	0.873423979	-7.885769395	4.446629884
1	0.621435250	-5.494854152	5.099540720
